# Disparities in nitrogen and phosphorus management across time and space: a case study of the Chesapeake Bay using the *CAFE* framework

**DOI:** 10.1088/1748-9326/ad786c

**Published:** 2024-09-25

**Authors:** Tan Zou, Eric A Davidson, Robert D Sabo, Graham K MacDonald, Xin Zhang

**Affiliations:** 1 Appalachian Laboratory, University of Maryland Center for Environmental Science, Frostburg, MD, United States of America; 2 Office of Research and Development, Center for Public Health and Environmental Assessment, U.S. Environmental Protection Agency, Washington, DC, United States of America; 3 Department of Geography, McGill University, Montreal, Quebec, Canada; 4 Global Nitrogen Innovation Center for Clean Energy and the Environment, Frostburg, MD, United States of America

**Keywords:** nutrient management, nutrient pollution, nitrogen reduction, phosphorus reduction, sustainability, sustainable agriculture

## Abstract

Efficient management of nitrogen (N) and phosphorus (P) is imperative for sustainable agriculture, resource conservation, and reducing environmental pollution. Despite progress in on-farm practices and urban wastewater treatment in the Chesapeake Bay (CB) watershed, limited attention has been given to nutrient transport, use, and handling between farms and urban environments. This study uses the hierarchical *CAFE* (Cropping system, Animal-crop system, Food system, and Ecosystem) framework to evaluate nutrient management performances within the watershed. We first develop a three-decade, county-level nutrient budget database (1985–2019), then analyze the spatiotemporal patterns of N and P budgets, as well as N and P use efficiencies, within the four *CAFE* hierarchies. Our results indicate a sizable increase in potential N and P losses beyond crop fields (i.e. in the Animal-crop system, Food system, and Ecosystem), surpassing losses from cropland in over 90% of counties. To address these system-wide trade-offs, we estimate the nutrient resources in waste streams beyond croplands, which, if recovered and recycled, could theoretically offset mineral fertilizer inputs in over 60% of counties. Additionally, the growing imbalance in excess N versus P across systems, which increases the N:P ratio of potential losses, could pose an emerging risk to downstream aquatic ecosystems. By utilizing a systematic approach, our novel application of the *CAFE* framework reveals trade-offs and synergies in nutrient management outcomes that transcend agro-environmental and political boundaries, underscores disparities in N and P management, and helps to identify unique opportunities for enhancing holistic nutrient management across systems within the CB watershed.

## 1. Introduction

Nitrogen (N) and phosphorus (P) are two key nutrients for agricultural production. However, inefficient use of these nutrients can lead to environmental pollution and resource scarcity problems (e.g. limited nutrient input to cropland in poor countries and a potential P scarcity problem in most countries) [1–5]. With large nutrient losses from agriculture, urban areas, and industries entering waterbodies, water quality issues and eutrophication problems have occurred, threatening ecosystem and human health around the world, including in the Chesapeake Bay (CB) of the United States [6–8].

The CB watershed spans parts of New York, Pennsylvania, Maryland, Virginia, West Virginia, Delaware and the District of Columbia [9]. Because the CB’s health and human activities are critically linked, the CB watershed has long been the focus of studies on nutrient management and pollution [6, 10–15]. To reduce nutrient pollution, the United States Environmental Protection Agency (USEPA) and CB states developed multiple phases of nutrient pollution reduction targets to reduce N loads to the CB to 215 million pounds and P loads to 13 million pounds by 2025 [16–19]. After years of efforts, some progress has been made [5, 15, 20, 21], but the nutrient load targets have not yet been achieved [20]. In addition, future climate and social changes, as well as the possible legacy effect of nutrients accumulating in the soil and groundwater [5, 17, 22], may offset improvements in nutrient management from adoption of best management practices in agriculture and from wastewater treatment plant upgrades [17, 23]. Other challenges to nutrient reduction include varied responses to practices by region [17, 21, 24] and discrepancies between results expected from modeling studies and actual measurements [20, 21].

Improvement in research and management practices is needed to achieve nutrient reduction goals and overcome challenges. For example, linking nutrient management among food production and consumption systems could reveal opportunities to reduce N and P losses across multiple sectors. Past research and nutrient reduction credits have focused on improving nutrient management in agriculture, decreasing emissions and deposition, attenuating urban non-point sources of nutrient pollution, and upgrading wastewater treatment, but with limited studies across these systems simultaneously [15, 25, 26]. In addition, there has been limited investigation into the balance between N and P management [27–30] and how N and P pollution sources are evolving through time and across sectors.

Here we employ the *CAFE* (Cropping system, Animal-crop system, Food system, and Ecosystem) framework [26, 31] to quantify nutrient budgets and efficiencies across a hierarchy of food production and consumption systems at the county level and to reveal trade-offs and synergies among different systems’ management outcomes. We also explore the theoretical potential for increasing nutrient recycling and transferring nutrients from regions and systems with an excess to regions that import nutrients. Lastly, we estimate the N:P surplus ratio to evaluate potential nutrient losses from each system and analyze the potential environmental consequences arising from nutrient imbalances.

## 2. Materials and methods

### 2.1. The *CAFE* framework and the CB watershed nutrient budget database

**Figure 1.**
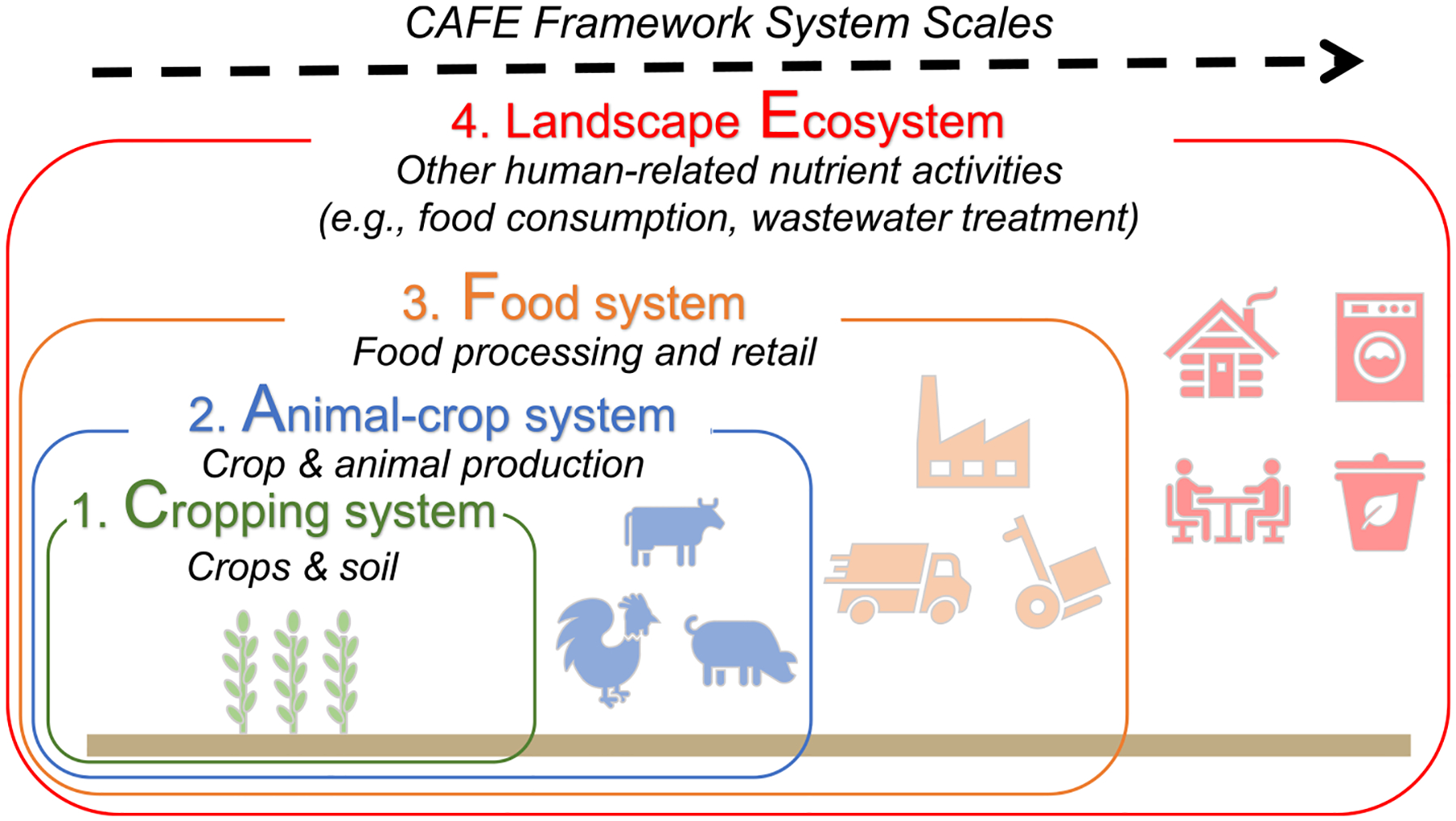
An illustration of the *CAFE* framework and the connected four systems within the framework. [26] John Wiley & Sons. ©2020 American Geophysical Union. All Rights Reserved.

The *CAFE* framework is composed of four nested, hierarchical nutrient management systems: CAFE (figure 1). Each system has multiple nutrient flows entering (inputs) or leaving (outputs) the system. Following this framework, we built an N and P budget database for 197 counties within the CB watershed by county, year, and system from 1985 to 2019, with data mainly from the Chesapeake Assessment Scenario Tool database, CAST-2019 [32], and the CB Nutrient Inventory [15]. Here we focus only on the major nutrient flows related to human production and consumption activities that can be quantified with currently available data for the CB watershed. Nutrient flows such as industrial production and pet food/waste are beyond the scope of this study but could be considered in future work [33, 34]. The loss of nutrients through leaching, runoff, or gaseous emissions are not separately quantified but are included as part of the nutrient surpluses calculated within the *CAFE* framework as described below. More details about the watershed background and location, the framework, and the calculations can be found in supplementary information (S1 and S2).

### 2.2. Nutrient management indicators

To evaluate the nutrient management performance of each system and region, commonly used indicators of nutrient use efficiency and nutrient surplus were employed [26]: nitrogen use efficiency (NUE), phosphorus use efficiency (PUE), N surplus, and P surplus.

Nutrient use efficiency is defined as the ratio of each system’s nutrient productive outputs (defined here as the outputs that can be used by humans and livestock, such as crop production) to the system nutrient inputs:

(1) $$\text{Nutrient}\;\text{use}\;\text{efficiency}=\frac{\text{system}\;\text{nutrient}\;\text{productive}\;\text{outputs}}{\text{system}\;\text{nutrient}\;\text{inputs}}.$$

System nutrient surplus is defined as the difference between a system’s nutrient inputs and the system’s nutrient productive outputs. The surpluses from all counties within the watershed are summed to calculate the total watershed-scale surplus. Nutrient surplus is a widely used indicator that represents the potential loss of nutrients as nutrient pollution if it is positive, or nutrients extracted from the system if it is negative [1–4]:

(2) Systemnutrientsurplus=systemnutrientinputs−systemnutrientproductiveoutputs.

Both nutrient use efficiency and nutrient surplus are discussed, as they reflect different aspects of nutrient management performance (see explanation in S2.5).

Changes in surpluses between systems and over time were then calculated. For example, the surplus change from Cropping system to Animal-crop system can be written as:

(3) $${\text{dN}}_{\text{sur},A,C,\text{co},\text{yr}}={\text{N}}_{\text{sur},A,\text{co},\text{yr}}-{\text{N}}_{\text{sur},C,\text{co},\text{yr}}$$

where dN_sur,*A*,*C*,co,yr_ is the N surplus change from Cropping system to Animal-crop system in county co and year yr, N_sur,*A*,co,yr_ is the Animal-crop system N surplus in county co and year yr, and N_sur,*C*,co,yr_ is the Cropping system N surplus in county co and year yr.

County level N and P surplus estimates (surplus divided by cropland area) were compared with thresholds of planetary boundaries (PBs) that serve as reference points for a safe range of agricultural surplus consistent with human and environmental health [35–41]. Based on thresholds for N deposition rates, N concentrations in surface water, and N concentrations in groundwater, PBs have been estimates as 24 kg N ha^−1^ yr^−1^ for global scale and 15 kg N ha^−1^ yr^−1^ for the United States [41]. The estimated global scale PB range for P has been estimated as 3.5–6.9 kg P ha^−1^ yr^−1^ [1, 40]. These boundaries were estimated through modeling using several assumptions, and they are used here only as points of reference.

**Figure 2.**
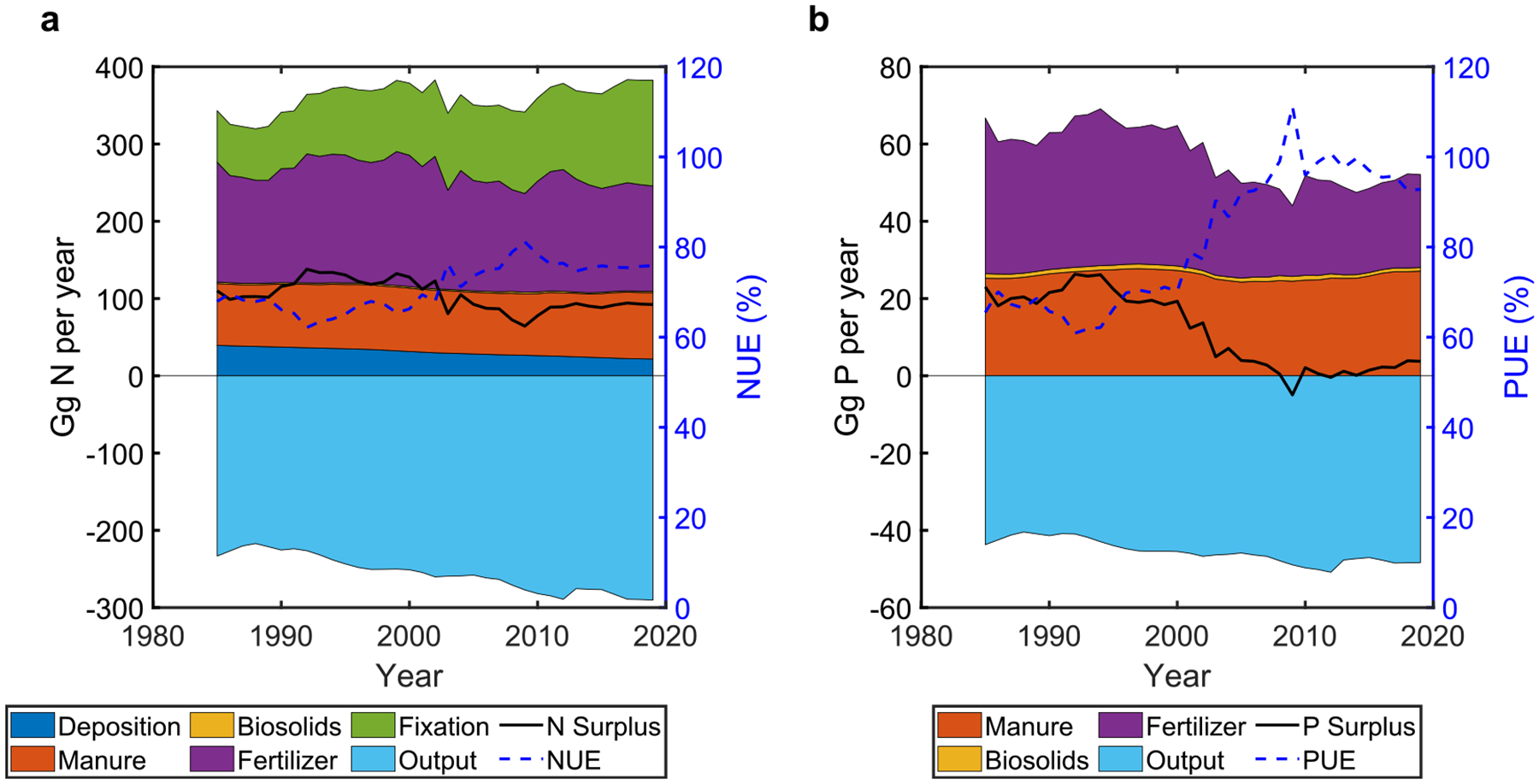
Watershed-scale cropland nutrient management performances. Cropping system N (a) and P (b) budget changes from 1985 to 2019 at the watershed scale. Positive values are inputs; negative values are Cropping system productive outputs (nutrients in harvested crops). Dotted lines represent NUE and PUE values; solid lines represent surplus values. PUE values >100% represent potential soil mining of P.

**Figure 3.**
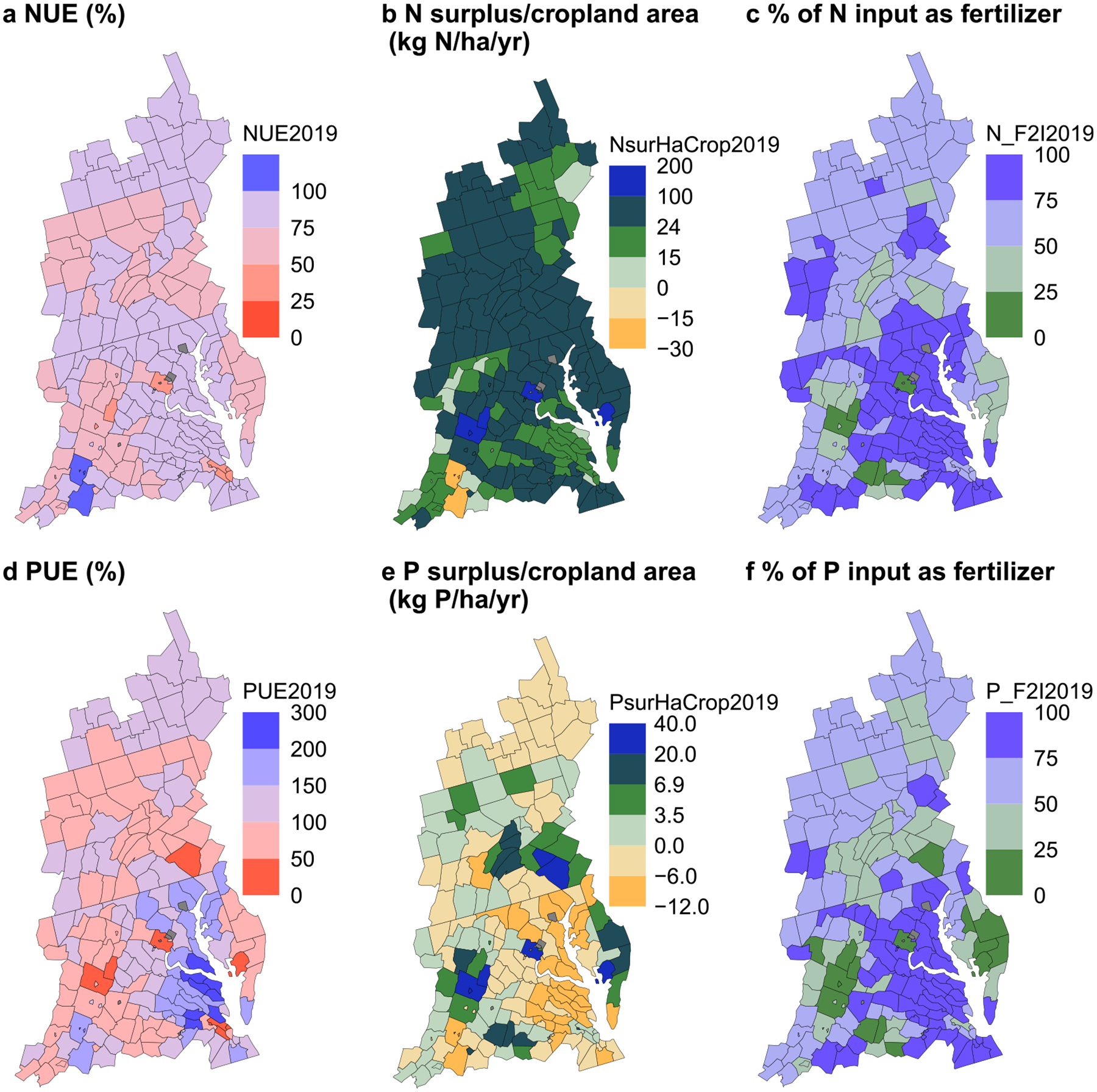
Cropland nutrient management performances in 2019 by county. Cropping system NUE (a) and PUE (d) (%). Cropping system N surplus (b) and P surplus (e) (kg N or kg P per ha cropland area per year). Cropping system portion of the sum of mineral fertilizer and manure as mineral fertilizer for N (c) and P (f) (%). Dark gray areas: no data. NUE and PUE values >100% represent potential soil mining. Figures for 1985 and changes between 1985 and 2019 can be found in Supplementary Information (figure S2). The surplus rates in panels b and e have the breaks at 15 and 24 kg N per ha cropland area per year for N and 3.5 and 6.9 kg P per ha cropland area per year for P to compare with the proposed planetary boundary safe ranges for human and environmental health [35–41]. The color scales vary among panels to virtually differentiate the different indicators and scales for each set of panels ((a) and (d), (b) and (e), (c) and (f)).

**Figure 4.**
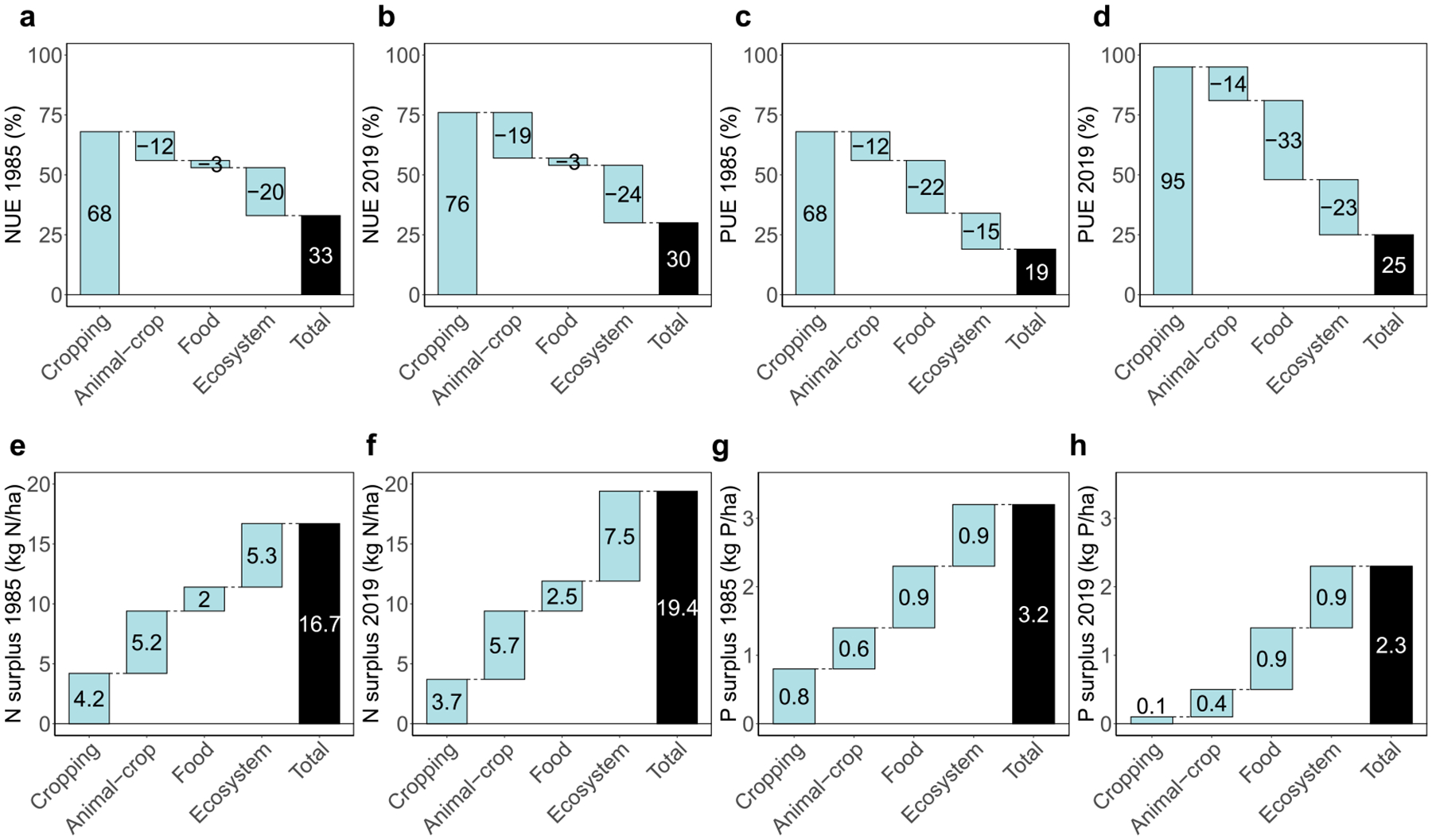
N and P efficiency and surplus changes across systems (i.e., value of the current system minus the value of the previous system) on the watershed scale. (a) NUE in 1985 (%). (b) NUE in 2019 (%). (c) PUE in 1985 (%). (d) PUE in 2019 (%). (e) N surplus in 1985 (kg N per ha watershed land area per year). (f) N surplus in 2019 (kg N per ha watershed land area per year). (g) P surplus in 1985 (kg P per ha watershed land area per year). (h) P surplus in 2019 (kg P per ha watershed land area per year).

**Figure 5.**
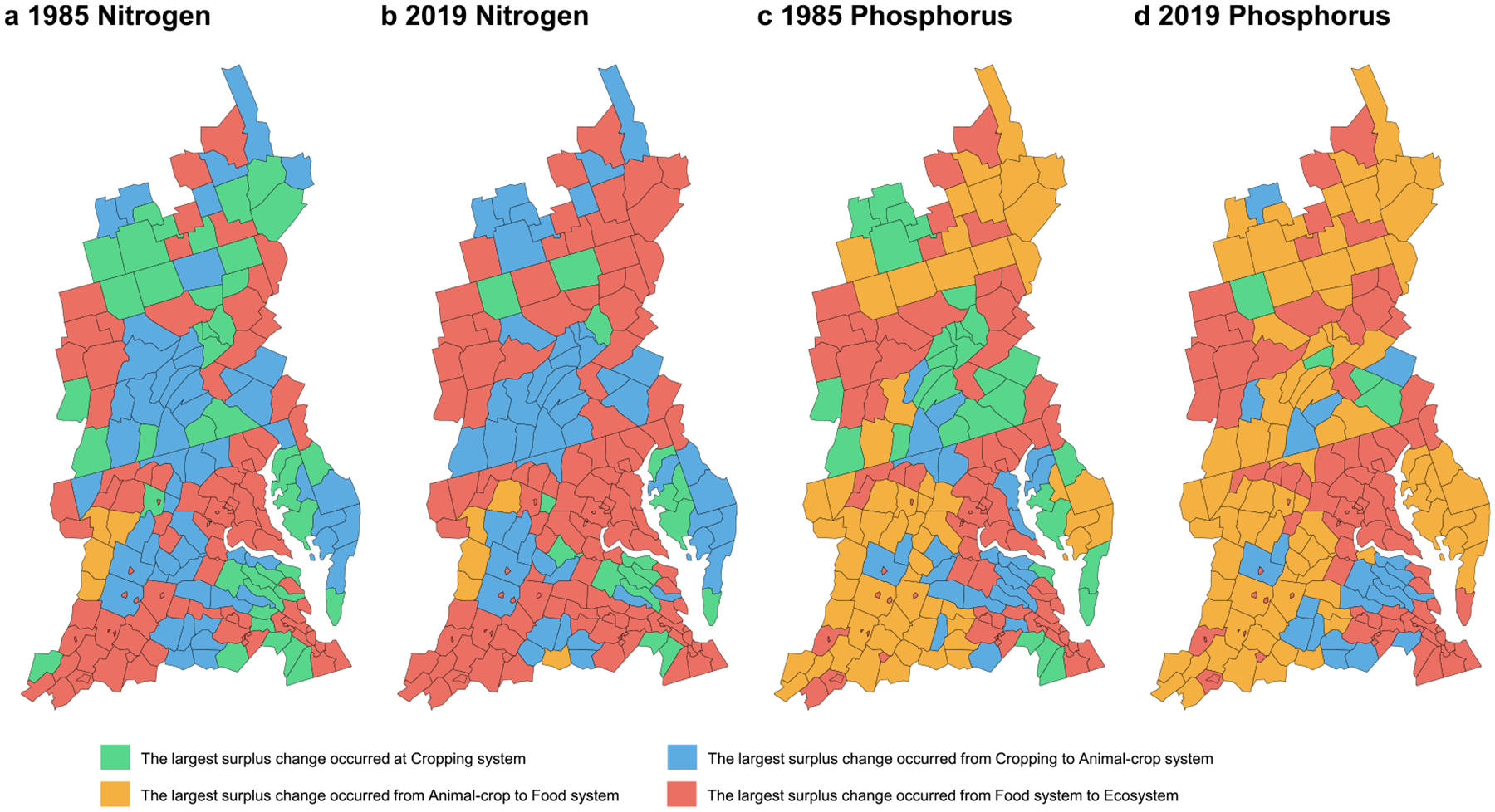
System with the largest positive surplus change in each county in 1985 (panel (a) for N, panel (c) for P) and 2019 (panel (b) for N, panel (d) for P). Surplus changes at the Cropping system reflect the exact values of the surpluses within this system. Only positive surplus changes are compared here, as positive surplus indicates potential nutrient loss. Surplus values by county can be found in figures 3 and S2.

**Figure 6.**
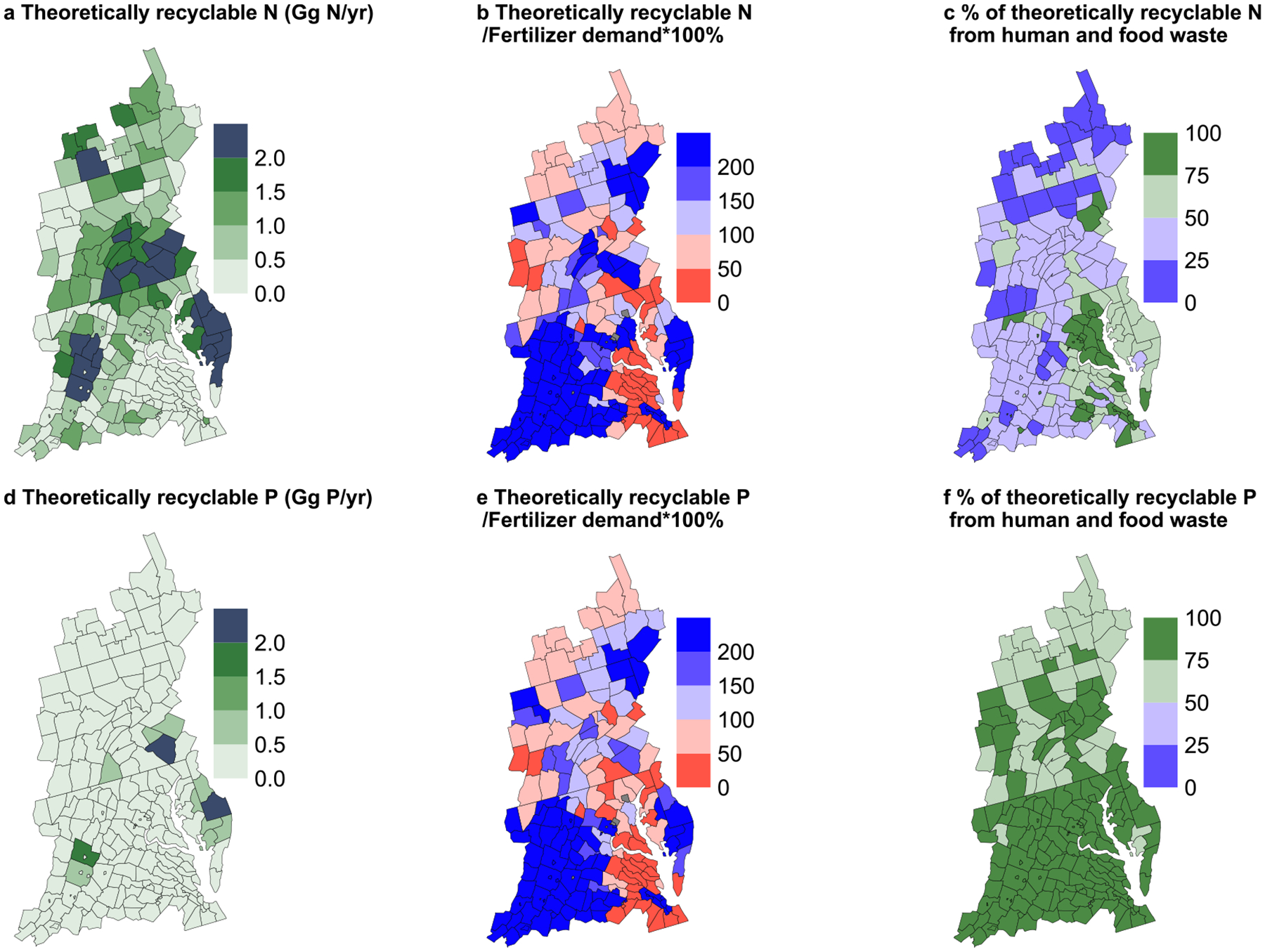
Theoretically recyclable nutrient amounts by county in 2019. Total theoretically recyclable N and P ((a) and (d), Gg N or Gg P per year). The percentage of local fertilizer demand that can be offset by total theoretically recyclable N or P in the same county ((b) and (e), %). The percentage of total theoretically recyclable N and P from human and food waste (versus unrecycled manure, panels (c) and (f), %). Spatial distribution in 1985 can be found in supplementary information (figure S8).

**Figure 7.**
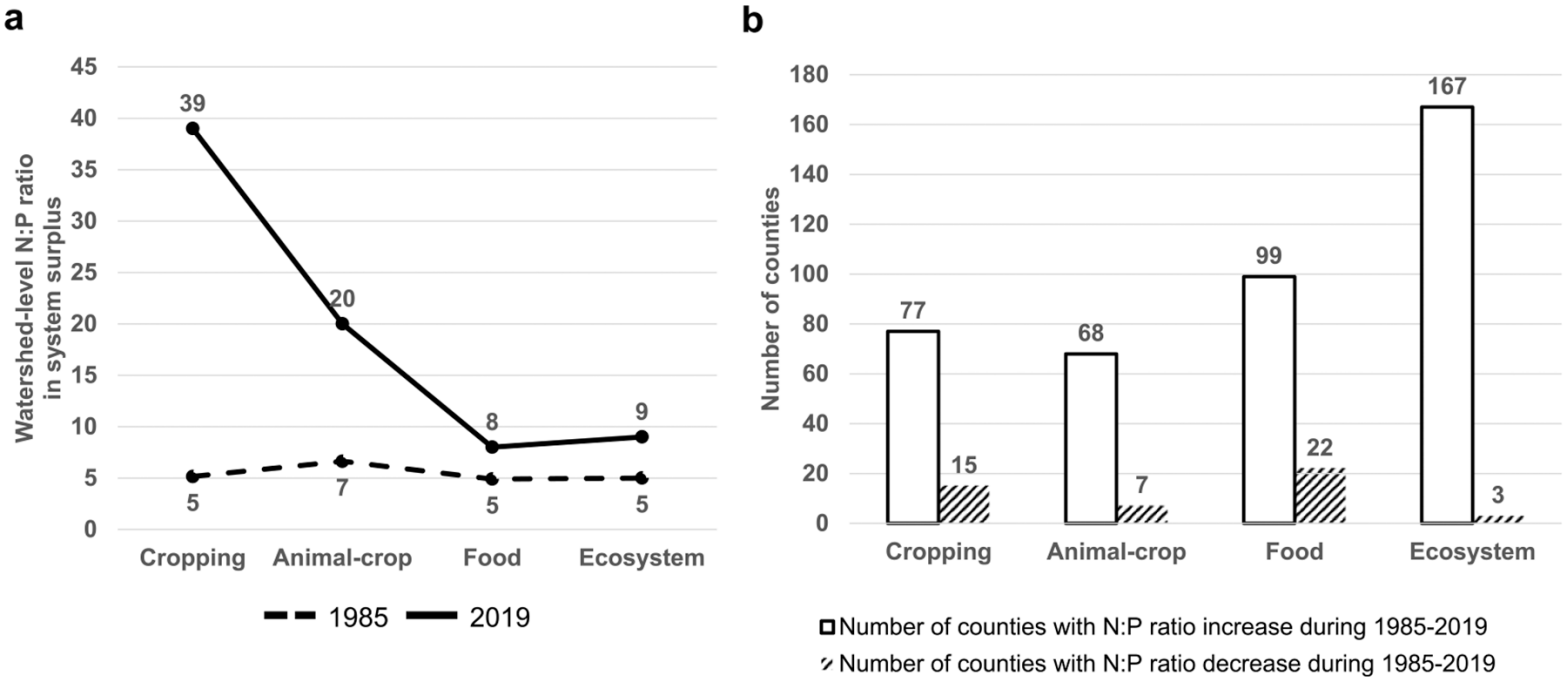
N:P ratios in system nutrient surpluses. (a) Watershed scale N:P ratios in system nutrient surpluses in 1985 and 2019. (b) Number of counties with N:P ratios in four systems increasing from 1985 to 2019 or decreasing from 1985 to 2019. N:P ratios by county across systems can be found in supplementary information (figure S13).

Comparisons of nutrient budget values for 1985 and 2019 are based on averages of the 1985–1989 and 2015–2019 periods, respectively, to smooth annual fluctuations (in the text, tables 1, 2 and figures 3–7). For the figure showing temporal changes across 35 years (figure 2), this averaging method is not conducted.

### 2.3. Total theoretically recyclable nutrients

Major types of nutrient waste include manure within the Animal-crop system, food waste from food processing and retail in the Food system, and human waste from the Ecosystem. Parts of these nutrient flows may be recycled or used for different purposes in the CB watershed, such as manure and biosolids application to soils for crop production (figure S7). Previous studies have defined and quantified nutrient waste from different sources across different spatial scales and time periods [42–44]. In this study, we define the total theoretically recyclable waste as the total amount of nutrients in wastes that can be quantified with currently available data but are not currently being recycled. It includes theoretically recyclable manure (i.e. currently unrecycled manure, but excluding manure left on pastures), theoretically recyclable food waste (i.e. waste generated from food processing and retail operations), and theoretically recyclable human waste (i.e. the sum of municipal wastewater treatment loads, industrial wastewater treatment loads, combined sewage overflows, and septic runoff, with data from CAST-2019 [15]). We also quantified the percentage of cropland mineral fertilizer input that could be ‘avoided’ by applying the total theoretically recyclable waste (for additional details, see S3). Although these potential nutrient sources represent a gradient of recoverability and feasibility for agronomic use (see section 4.2 for discussion), we include them here to provide a consistent and comprehensive accounting of the potential for nutrients from different waste streams to help offset mineral fertilizer use in the CB.

### 2.4. Statistical analyses

To compare the medians of more than two groups of data and determine if they are statistically different, the Kruskal–Wallis test was applied [45]. This is a nonparametric test that does not require a normal distribution of data. To examine the relationship between nutrient use efficiency and nutrient fertilizer input, data from 2019 were grouped into five groups of counties based on their percentage of N or P inputs as fertilizer: 0%–20%, 20%–40%, 40%–60%, 60%–80%, and 80%–100% (see figures S9(c) and (d)). We then compared the N or P inputs as fertilizer with the NUE or PUE distributions of the counties within each group for 2019. Similarly, to examine the relationship between county-level soil plant-available P (Mehlich 3, Bray or Mehlich 1, see S2.1) and PUE in 2019, as well as the relationship between county-level soil plant-available P and P fertilizer input, counties were categorized into six groups based on their 2019 PUE: 0%–20%, 20%–40%, 40%–60%, 60%–80%, 80%–100%, and ⩾100% (figure S11(c)), and five categories based on their percentage of P inputs as fertilizer: 0%–20%, 20%–40%, 40%–60%, 60%–80%, and 80%–100% (figure S11(d)). Linear regression was also applied to study the relationship between different variables.

### 2.5. Nitrogen-to-phosphorus ratio in surplus

The N:P ratio in surplus is defined as the ratio of N surplus to P surplus in each system. Negative surpluses are not included here since the focus is on the potential to reduce nutrient loading to the CB.

## 3. Results

### 3.1. Improved cropland nutrient management and decreased dependence on external inputs

Nutrient management in croplands of the CB watershed has improved since 1985, as evidenced by increased nutrient use efficiencies and decreased nutrient surpluses (figure 2, table 1). Cropping system NUE at the watershed scale increased from 68% in 1985% to 76% 2019, respectively, and PUE increased from 68% to 95%, respectively (table 1). Additionally, basin-wide N and P surpluses decreased from 103 to 92 Gg N yr^−1^, and from 20 to 3 Gg P yr^−1^, respectively, which is a reduction of 11% and 85% for N and P surplus, respectively.

The Cropping system N surplus rate (calculated as N surplus divided by cropland area) at the watershed scale in 1985 was estimated at 41 kg N ha^−1^ yr^−1^ and remained relatively stable in 2019 to 40 kg N ha^−1^ yr^−1^. The CB watershed-scale P surplus decreases from about 8.1 kg P ha^−1^ cropland area yr^−1^ in 1985 to 1.2 kg P ha^−1^ cropland area yr^−1^ in 2019. However, when normalizing by total watershed land area, which better represents potential for nutrient export from the whole watershed, the surpluses decreased from 4.2 to 3.7 kg N ha^−1^ yr^−1^, and from 0.82 to 0.11 kg P ha^−1^ yr^−1^. A reduction in P inputs and increases in N and P productive outputs (nutrients in harvested crops) resulted in improved NUE and PUE and decreased basin-wide N and P surpluses (figure 2, table 1).

At the county scale, both NUE and PUE increased and N and P surpluses decreased in many counties, and most counties’ nutrient use efficiencies are already at a relatively high level (figures 3 and S2). In 2019, 96% of counties had a NUE exceeding 50%, and 95% of counties had a PUE exceeding 50%. Notably, 57% of counties exhibited a PUE exceeding 100%, indicating potential soil mining in these counties in recent years, most likely of legacy P from previous years’ fertilization. However, when aggregated at the watershed level, the P surplus was still positive in 2019 and in most years from 1985 to 2019 (figure 2(b)). Counties with higher NUE and PUE were typically found in New York and Maryland. Conversely, certain counties, such as Somerset in Maryland, Lancaster in Pennsylvania, and a few counties in Virginia, displayed relatively lower NUE or PUE, coupled with larger N or P surpluses. These counties were associated with varying levels of cropland production density (i.e. crop production in kg N or kg P per county area in ha, see figure S5). A comparative analysis of nutrient use efficiency and surplus changes between 1985 and 2019 (figure S2) revealed that 84% and 91% of counties experienced an increase in cropland NUE and PUE, respectively. A decline in cropland N and P surplus (normalized by cropland area) occurred in 75% and 92% of counties, respectively. The N surplus rates (surplus divided by cropland area) of the watershed and for most of the 197 counties in the CB watershed exceeded both the estimated global PB reference point (128 counties exceeded 24 kg N ha^−1^ yr^−1^) and the point for the regional scale PB for the U.S. (175 counties exceeded 15 kg N ha^−1^ yr^−1^) in 2019 (figure 3). The P surplus at the watershed level and in most counties decreased in recent decades to values lower than the P boundaries (189 counties with P surplus not exceeding 6.9 kg P ha^−1^ yr^−1^ in 2019).

The primary sources of human-introduced inputs for both N and P over the past 35 years were mineral fertilizers and manure (figure 2). From 1985 to 2019, the percentage of total cropland inputs as mineral fertilizer at the watershed scale (i.e. mineral fertilizer input/(mineral fertilizer input + manure input)*100) remained at about 62% for N, but decreased from 58% to 46% for P, suggesting that the dependency of row crop nutrient inputs on P fertilizer had decreased. Decreased dependence on fertilizer inputs was found for P at the county scale (figures S2 and 3). In 1985, 82% and 76% of counties depended more on fertilizer input than manure at the Cropping system for N and P, respectively; these percentages increased to 86% for N and decreased to 68% for P in 2019. The spatial distribution of the proportions of N and P input as fertilizer were very similar (figures S2 and 3).

A county’s cropland dependence on mineral fertilizer or recycled manure in the Cropping system is related to its Cropping system NUE and PUE. Comparing the Cropping system NUE and PUE by county and Cropping system portion of total inputs as fertilizer (figure 3), counties with a larger portion of cropland input from mineral fertilizer tended to have a higher NUE and PUE. Statistical analyses also identified a positive and statistically significant relationship (*p*-value < 0.05) between the N percentage of input from fertilizer and NUE, as well as between the percentage of P inputs from fertilizer and PUE (figure S9).

The linear regression and the Kruskal–Wallis test results (figure S11) also revealed a strong negative correlation (*p*-value < 0.05) between the proportion of cropland P input from fertilizer and the current soil plant-available P level, implying a positive relationship between the proportion of cropland P input from manure and soil plant-available P. These results suggest that regions currently with higher reliance on recycled manure for cropland inputs (figure 3) may have a greater amount of legacy P accumulation that can be utilized by crops (figure S3), which can be partly explained by the higher livestock density and thus the greater availability of recyclable manure in or near these regions (figure S5).

### 3.2. Increased nutrient surpluses beyond croplands

While the Cropping system produced a large amount of nutrient surplus, systems beyond croplands made an even greater contribution to nutrient surpluses in the CB watershed. Comparing nutrient surpluses across systems, the annual N surplus and P surplus per ha watershed land area increased when transitioning from the Cropping system to the Animal-crop system, Food system, and Ecosystem in both the 1980s and 2010s (figure 4). However, when comparing nutrient surpluses across different time periods, the N surpluses at the Cropping system and Animal-crop system did not change much from 1985 to 2019 and the P surpluses at the two systems significantly decreased from 1985 to 2019, but the N surplus at the Food system and Ecosystem increased from 1985 to 2019. These temporal changes, varying across systems, can be partly attributed to the following changes from 1985 to 2019: (1) slightly improved NUE at the Cropping and Animal-crop systems from 1985 to 2019 but much larger improvement of PUE during the same period, (2) growth in the watershed population (figure S6), and (3) a net increase in the nutrient import rate (i.e. nutrients in imported feed and food/system nutrient inputs*100%) relative to the nutrient export rate (i.e. exported nutrients/system nutrient productive outputs*100%) within the Food system (figure S6). As a result, the Cropping system’s N and P surpluses only accounted for 40% and 23% of the Animal-crop system’s N and P surpluses, 31% and 8% of the Food system’s surpluses, and 19% and 5% of the Ecosystem’s surpluses, respectively, in 2019 (figure 4).

Of the 197 counties within the CB watershed in 1985, 53%, 65%, and 92% had Cropping system N surpluses smaller than half of the surpluses at Animal-crop system, Food system, and Ecosystem, respectively (table 2). This means that for over half of the counties, N surpluses beyond croplands was larger than on-farm N surpluses. And in 2019, the percentages of counties that had Cropping System N surpluses smaller than half of surpluses at Animal-crop System, Food System, and Ecosystem were 52%, 63%, and 97%, respectively, with only 1%–5% change from 1985. Similarly, a large portion of counties had P surpluses beyond croplands larger than on-farm P surpluses (47%, 77%, and 91% in 1985; 63%, 93%, and 96% in 2019), and the percentage of counties with larger P surpluses beyond croplands increased from 1985 to 2019. Comparing the surplus changes between systems in 2019 (figure 5), the increase in surpluses was more pronounced when transitioning from the Food system to the Ecosystem. Approximately 64% and 46% of all counties exhibited the greatest positive changes in N and P surplus, respectively, when moving from the Food system to the Ecosystem, compared to surplus changes between other interconnected systems. This suggests an increase in nutrient losses as human waste. These patterns can be partly explained by a large and increasing human population density in these regions (figure S6). Discussions on NUE and PUE changes can be found in supplementary information (S2.6).

### 3.3. Large theoretically recyclable waste beyond croplands

By comparing total theoretically recyclable waste and mineral fertilizer demand for each county, we identified counties with total theoretically recyclable nutrients larger than local mineral fertilizer demand (here defined as potential nutrient sources) and counties with theoretically recyclable nutrients smaller than mineral fertilizer demand (here defined as potential nutrient sinks). At the watershed scale, total theoretically recyclable waste decreased slightly from 1985 to 2019 for both N (217 Gg N in 1985–210 Gg N in 2019) and P (35 Gg P in 1985–32 Gg P in 2019). However, in 2019, the total theoretically recyclable waste was still large enough that it could, in principle, be diverted to offset all local mineral fertilizer demand for both N (137 Gg N) and P (23 Gg P) in the CB watershed, with some leftover.

The theoretically recyclable waste could help avoid a large proportion of the mineral fertilizer use in croplands in many counties (figure 6). Around 62% and 55% of 197 counties in 1985 had total theoretically recyclable waste that could have been diverted to avoid all fertilizer N and P inputs, respectively. These ratios increased to 66% and 62% in 2019, respectively. This means that more than 60% of all counties had total theoretically recyclable wastes large enough to cover all mineral fertilizer inputs if all unused nutrient from manure production, food processing, food retail, and human consumption could be recycled. Regions with higher livestock density (figure S5) or higher human population density (figure S6) often generated larger amounts of theoretically recyclable waste. Unrecycled manure stands out as the primary source for N (figure 6(c)), with approximately 56% of counties having more than half of their N in total theoretically recyclable waste originating from unrecycled manure. In contrast, for P (figure 6(f)), all counties have over 50% of their total theoretically recyclable waste coming from food and human waste. These patterns can be partly explained by large N losses through gas emissions in manure [3, 46, 47], the relatively higher ratio of P loss from the Food system compared to N (table S6), and the increasing or already relatively large population densities in these regions (figure S6).

### 3.4. Increased N:P ratios in the system nutrient surpluses

In the CB watershed, the N:P ratios of system nutrient surpluses have increased from 1985 to 2019, which may lead to a large nutrient imbalance in nearby waterbodies (figure 7). In 1985, the N:P surplus ratios were 5, 7, 5, and 5 from the CAFE respectively. In contrast, the N:P surplus ratios increased to 39, 20, 8, and 9 for the four systems in 2019. These changes in N:P ratios at the Cropping and Animal-crop systems were primarily due to a much larger reduction in P surplus than that of N surplus over time (figure 2, table 1). At the county-scale, a large number of counties had an increasing N:P ratio from 1985 to 2019 in all four systems (77, 68, 99, and 167, respectively), especially at the Ecosystem level, whereas only a few counties had a decreasing ratio from 1985 to 2019 in all four systems (15, 7, 22, and 3, respectively).

## 4. Discussion

### 4.1. Improving nutrient management beyond farms and farmers with the *CAFE* framework

Applying the *CAFE* framework to the CB watershed identified nutrient management gaps and opportunities across different systems. Nutrient use efficiencies and surpluses were quantified and compared across four different food production and consumption stages. This allows us to identify the stage(s) with a relatively higher nutrient loss potential or/and relatively lower nutrient use efficiency compared to other systems by region, and inform policymakers about prioritizing efforts to reduce the nutrient loss or/and improve the nutrient use efficiency from these stages.

For nutrient management performance at the Cropping system level, both reduced surpluses and improved nutrient use efficiencies align with the findings in previous watershed nutrient studies [5, 15, 22]. These temporal variations in efficiencies and surpluses during 1985–2019 are the result of many factors, such as long-term production patterns and a series of social, policy, and strategy changes (see more discussions in SI S2.6). The drops in N and P surpluses and mineral fertilizer inputs in 2009 coincided with a series of socioeconomic and agricultural changes, such as the economic recession in the U.S. starting in December 2007 [48], the spike in global fertilizer prices during 2007–2010 [49, 50], and the decrease in fertilizer use in the U.S. in 2009 [51, 52].

Comparing nutrient management performances across systems, our results show that croplands contribute only a small fraction of the overall nutrient loss potential, and that nutrient use efficiency declines from the Cropping system to the Ecosystem. The largest increase in N surplus occurred between the Food system and the Ecosystem. This increase was attributed to excess N potential loss in the Ecosystem compared to the Food system, arising from urban fertilizer use, urban deposition and fixation, and food and human waste (figure S1, table S3). Therefore, an effective strategy for further reducing the overall N surplus involves upgrading wastewater treatment plants to further reduce N discharge from human waste, and enhancing its recycling for urban and rural agricultural use.

In contrast, large P surplus increases were observed between the Animal-Crop system and the Food system, and between the Food system and the Ecosystem. The additional potential P loss from the Animal-Crop system to the Food system was primarily due to losses from food processing and retail (figure S1, table S4), such as nutrient losses in inedible portions during beef production, where 89.2% of P was lost, compared to 54.4% loss of N (table S6). Therefore, reducing the overall P surplus also requires improvements in nutrient management within food processing and retail sectors.

At the county-scale, the potential nutrient loss from animal production, food processing and retail, and human consumption surpasses that of crop production in an increasing number of counties within the CB watershed, for both N and P. These patterns suggest that in addition to current practices on farms and upgrading wastewater treatment, other strategies also need to be implemented to further reduce and recycle nutrient losses from non-point sources and from waste that ends up in landfills or is incinerated [31, 53, 54]. Diverting food waste from landfills would have the added benefit of reducing methane emissions [55]. Applying these strategies requires engaging a diverse group of stakeholders (e.g. policy makers, food and transport industries, treatment facility operators, and consumers), beyond only farmers, in efforts to improve nutrient management and reduce nutrient surplus. Strategies to improve nutrient management in each system can be found in supplementary information (S2.7).

### 4.2. Addressing trade-offs and promoting synergies of nutrient management outcomes across the *CAFE* systems

The *CAFE* framework also reveals the complex interconnections among different nutrient management systems, and synergies and trade-offs among the nutrient management outcomes of all systems. This interrelatedness presents challenges, but also opportunities for synergistic nutrient management, which may be more effective than management focused only on one system.

One important strategy to address trade-offs and promote synergies is to increase the recycling of nutrients between systems and among regions, while ensuring that nutrients from mineral fertilizer and other sources are used judiciously in agriculture to avoid excess nutrient inputs. Our study has revealed a large potential for coupling systems to meet local cropland nutrient demands with local nutrient surpluses. We found that nutrients in local unrecycled manure, food waste, and human waste could theoretically offset the current mineral fertilizer inputs to croplands in over 60% of counties, as well as at the CB watershed scale (section 3.3, figure 6). Regions with a higher livestock density (figure S5) and/or human density (figure S6) are usually the hot spots of these wastes [42, 44, 56]. The uneven distribution of theoretically recyclable nutrients also indicates the need for economically viable options to process and transport nutrients across county boundaries from nutrient sources to sinks (figure 6). Recylable nutrients from animal, food waste, and human waste could also be applied to urban and suburban landscaping and gardening by urban gardeners and farmers, possibly encouraged with funding support from NGOs or government agencies.

However, our estimate of theoretically recyclable waste is hypothetical. It is important to recognize that technical, logistical, market, and environmental challenges exist, which also explain that currently only small fraction of cropland inputs are from recycled biosolids (figure 2). Discussions on these challenges and how to address them can be found in supplementary information (S3). In addition, this study provides an ideal scenario, exploring all theoretical possibilities for reusing waste. Using the *CAFE* framework and data synthesized in this study, future researchers can customize the calculations and study the locally recyclable total nutrient amounts based on known local conditions (e.g. the extent of pasture fertilization, supplemental feed, or the wastewater treatment technologies) in the CB watershed and other regions.

At the same time, reducing nutrient loss from food processing and retail, and promoting diets with a smaller nutrient footprint may present viable options for improving nutrient management. Fortunately, efforts are already underway to overcome these challenges and barriers, with some successful examples (supplementary information S3). For instance, the U.S. Department of Agriculture and the USEPA have announced the U.S. 2030 Food Loss and Waste Reduction goal, which aims to halve food loss and waste by 2030 compared to the baseline year of 2016 [57]. In addition, the concept of ‘manuresheds’ has been proposed to link counties with surplus manure nutrients to those with nutrient demands, taking into account factors such as the N:P ratio in manure, the distance between counties, the availability of manure nutrients, and the demand for nutrients [43].

### 4.3. Unbalanced nitrogen and phosphorus surpluses

The N:P surplus ratio at the watershed-scale increased for each system after three decades since 1985, mainly due to a larger decrease in P surplus compared to N surplus (figure 2). Other studies of different spatial scales have also found this increasing trend of N:P surplus ratios [3, 58]. The nutrient surpluses calculated in this study do not necessarily indicate the nutrients actually delivered to the CB, because a fraction of both N and P surpluses could be retained in soils, vegetation, and accumulate in groundwater, and N surpluses can also be lost in gaseous forms. Nevertheless, it is worth noting that these changes in N:P ratios of surpluses at country and watershed scales could be indicative of changing pressures on the Bay. Some phytoplankton species adapted to high N:P ratios can cause harmful algal blooms and disruption to the aquatic food webs [58–60]. Excess N has been a long-standing problem in the CB watershed, leading to algal blooms and hypoxia [61, 62] and N has been the primary goal for reduction in the CB Total Maximum Daily Load (TMDL) [63]. Our findings highlight the need for improved N management to control the amount of N surplus lost to the environment while still controlling P loss, and to prevent unwanted consequences of high N:P ratios of surpluses. N:P surplus ratios could further increase due to high N:P ratio in mineral fertilizer, reduced or banned use of P in detergents, high inputs of N to the Bay, improved PUE across systems, and more efficient removal of P in the human waste by upgraded wastewater treatment plants [58].

### 4.4. Uncertainty and limitations

There is a lack of comprehensive data to construct all of the components of the county-scale and annual nutrient budget database for the last three decades. Consequently, certain assumptions and fixed parameters had to be employed in the estimation process. For example, limited data for long-term and annual amounts and sources of imported nutrients (imported fertilizers, human food, and animal feed) and destinations of exported nutrients (within or beyond the CB watershed) make it hard to quantify the nutrients embedded in trade. Therefore, we inferred the magnitude of these trade-related flows based on assumptions of mass-balance (i.e., production = local use + import–export). Another example is determining the portion of nutrients in agricultural products that are produced for human consumption (edible portion) and the portion for non-food uses or other losses and waste (inedible portion). Definitions and parameters were chosen based on previous studies to estimate nutrient amounts for different usages. More details about our assumptions and the parameters we used can be found in supplementary information (S1, S2.1–2.4 and S6).

The nutrient flow estimates we collected from public datasets and parameter data we synthesized from the literature sources are a combination of monitored data and modeled results. Some parameter data were measured in specific regions and at specific times, which may not fully capture temporal changes or spatial variations in reality. Additionally, as the available budget data are limited to the period between 1985 and 2019, this study is unable to examine historical nutrient usage patterns prior to 1985. To address these uncertainties and limitations, future research efforts can focus on better monitoring and recording the quantity and pathways of nutrients with the *CAFE* framework and across the four systems.

## 5. Conclusions

By studying the historical and spatial patterns of nutrient use, we found that management gaps in the CB watershed vary by nutrient type, spatial scale, region, system, and time. The large potential nutrient loss beyond croplands suggests the importance of further improving nutrient management within the Food system and Landscape Ecosystem under the *CAFE* framework, in addition to the Cropping and Animal-crop systems. The trade-offs among nutrient management outcomes across systems and across regions require further attention and actions, including: (1) improving monitoring of nutrient use efficiencies and surpluses across the four *CAFE* systems; (2) implementing strategies to achieve nutrient management improvement; (3) increasing the recovery and recycling of theoretically recyclable waste onto croplands and for animal feed, both within and across regional boundaries.

Our findings also highlight an increasing N:P ratio in surplus in the past three decades in the CB watershed. The N:P ratio in nutrient surplus serves as a useful benchmark to assess possible nutrient imbalances. In addition, the implications of the N:P ratio and the nutrient surpluses for agricultural production and environmental health need to be explored on a region-by-region basis.

Finally, our study suggests that a holistic approach to nutrient management, like the *CAFE* framework, which considers not only on-farm management but also management beyond croplands, is important for achieving sustainable nutrient management from food production to consumption and for identifying opportunities to systematically reduce nutrient waste. The findings resulting from the application of this framework can be discussed among diverse stakeholders and then serve as the foundation for developing plans and strategies aimed at achieving synergies among different sustainability and socioeconomic goals through collaborative efforts. Besides the CB watershed case study, our approach can be applied to other regions and spatial scales to enhance their nutrient management. At the same time, more studies are needed to improve the availability and accuracy of nutrient use and loss data at finer spatial scales and to address the socioeconomic and technical barriers faced by communities to improve nutrient recovery and recycling.

## Supplementary Material

Supplement1

Supplement2

## Figures and Tables

**Table 1.** Summary of watershed-scale nutrient budgets across the four *CAFE* systems. NUE: nitrogen use efficiency; PUE: phosphorus use efficiency.

Year and nutrient type	Item/system	Cropping system	Animal-crop system	Food system	Ecosystem
1985 Nitrogen	System input (Gg N yr^−1^)	327	526	593	616
	System productive output (Gg Ny r^−1^)	224	294	315	206
	System nutrient surplus (GgNyr^−1^)	103	232	279	410
	System NUE (%)	68	56	53	33
2019 Nitrogen	System input (Gg N yr^−1^)	378	540	638	681
	System productive output (Gg N yr^−1^)	286	310	346	204
	System nutrient surplus (Gg N yr^−1^)	92	230	292	477
	System NUE (%)	76	57	54	30
1985–2019 Nitrogen budget changes	System input in 2019 minus System input in 1985 (Gg N yr^−1^)	51	14	45	65
	System productive output in 2019 minus System productive output in 1985 (Gg N yr^−1^)	62	16	31	−2
	System nutrient surplus in 2019 minus System nutrient surplus in 1985 (Gg N yr^−1^)	−11	−2	13	67
	System NUE in 2019 minus System NUE in 1985 (%)	8	1	1	−3
1985 Phosphorus	System input (Gg P yr^−1^)	62	80	86	98
	System productive output (Gg P yr^−1^)	42	45	30	18
	System nutrient surplus (Gg P yr^−1^)	20	35	57	79
	System PUE (%)	68	56	34	19
2019 Phosphorus	System input (Gg P yr^−1^)	51	59	68	73
	System productive output (Gg P yr^−1^)	48	48	33	18
	System nutrient surplus (Gg P yr^−1^)	3	12	36	55
	System PUE (%)	95	81	48	25
1985–2019 Phosphorus budget changes	System input in 2019 minus System input in 1985 (Gg P yr^−1^)	−11	−21	−18	−25
	System productive output in 2019 minus System productive output in 1985 (Gg P yr^−1^)	6	3	3	0
	System nutrient surplus in 2019 minus System nutrient surplus in 1985 (Gg P yr^−1^)	−17	−23	−21	−24
	System PUE in 2019 minus System PUE in 1985 (%)	27	25	14	6

**Table 2.** Comparison of watershed-scale nutrient use efficiencies and surpluses across the four *CAFE* systems.

Year and nutrient type	1985 Nitrogen	2019 Nitrogen	1985 Phosphorus	2019 Phosphorus
% of counties with Cropping system nutrient surplus less than half of its Animal-crop system’s nutrient surplus	53%	52%	47%	63%
% of counties with Cropping system nutrient surplus less than half of its Food system’s nutrient surplus	65%	63%	77%	93%
% of counties with Cropping system nutrient surplus less than half of its Ecosystem’s nutrient surplus	92%	97%	91%	96%
% of counties with Cropping system nutrient use efficiency higher than its Animal-crop system’s nutrient use efficiency	61%	62%	64%	49%
% of counties with Cropping system nutrient use efficiency higher than its Food system’s nutrient use efficiency	55%	57%	80%	76%
% of counties with Cropping system nutrient use efficiency higher than its Ecosystem’s nutrient use efficiency	85%	92%	97%	91%

## Data Availability

All data that support the findings of this study are included within the article (and any supplementary files).
