# Supplementary material for: Disparities in nitrogen and phosphorus management across time and space: a case study of the Chesapeake Bay using the CAFE framework
Source: Environ Res Lett. Author manuscript; Available in PMC 2025 Sep 25. (PMC11977706; doi:10.1088/1748-9326/ad786c)
Supplement: Supplement2 [file NIHMS2052799-supplement-Supplement2.docx]

Supplementary Information

**Disparities in nitrogen and phosphorus management across time and space: a case study of the Chesapeake Bay using the *CAFE* framework**

AUTHORS

Tan Zou^1*^, Eric A Davidson^1,4^, Robert D Sabo^2^, Graham K MacDonald^3^ and Xin Zhang^1,4*^

^1^ Appalachian Laboratory, University of Maryland Center for Environmental Science, Frostburg, MD, United States of America

^2^ Office of Research and Development, Center for Public Health and Environmental Assessment, U.S. Environmental Protection Agency, Washington, DC, United States of America

^3^ Department of Geography, McGill University, Montreal, Quebec, Canada

^4^ Global Nitrogen Innovation Center for Clean Energy and the Environment, Frostburg, MD, United States of America

* Corresponding authors:

Tan Zou: [tan.zou@umces.edu](mailto:tan.zou@umces.edu)

Xin Zhang: [xin.zhang@umces.edu](mailto:xin.zhang@umces.edu)

DISCLAIMER: The views presented here are those of the authors and do not represent official views or policy of the U.S. Environmental Protection Agency or any other U.S. federal agency. Any use of trade, firm, or product names is for descriptive purposes only and does not imply endorsement by the U.S. Government.

Table of Contents

[S1 The *CAFE* framework 3](#_Toc175150310)

[S2 The Chesapeake Bay watershed nutrient budget database 4](#_Toc175150311)

[S2.1 Cropping system 4](#_Toc175150312)

[S2.2 Animal-crop system 8](#_Toc175150313)

[S2.3 Food system 11](#_Toc175150314)

[S2.4 Ecosystem 13](#_Toc175150315)

[S2.5 Discussing both nutrient use efficiency and nutrient surplus 14](#_Toc175150316)

[S2.6 Nutrient use efficiency and surplus changes across the time 14](#_Toc175150317)

[S2.7 Strategies to improve nutrient management in each system 15](#_Toc175150318)

[S3 Theoretically recyclable waste 15](#_Toc175150319)

[S4 Statistical analysis 18](#_Toc175150320)

[S5 Nitrogen to phosphorus ratio in surplus 21](#_Toc175150321)

[S6 Definition and parameters 23](#_Toc175150322)

[References 35](#_Toc175150323)

# S1 The *CAFE* framework

The *CAFE* framework developed in the previous work ^1,2^ is composed of four connected nutrient management systems (Cropping system, Animal-crop system, Food system, and Ecosystem) in a hierarchical manner. Major nitrogen (N) and phosphorus (P) nutrient flows in this study entering (inputs) or leaving the system (outputs) are illustrated below.


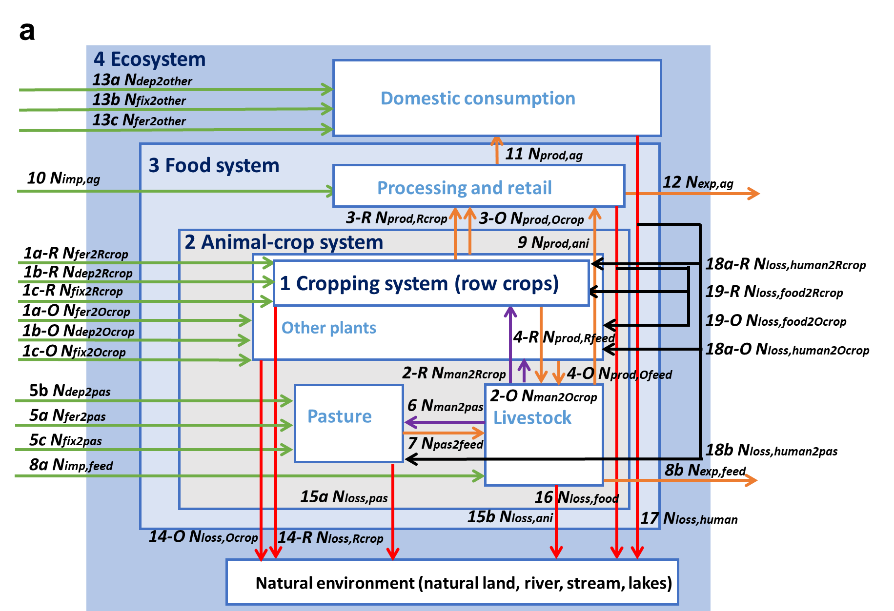

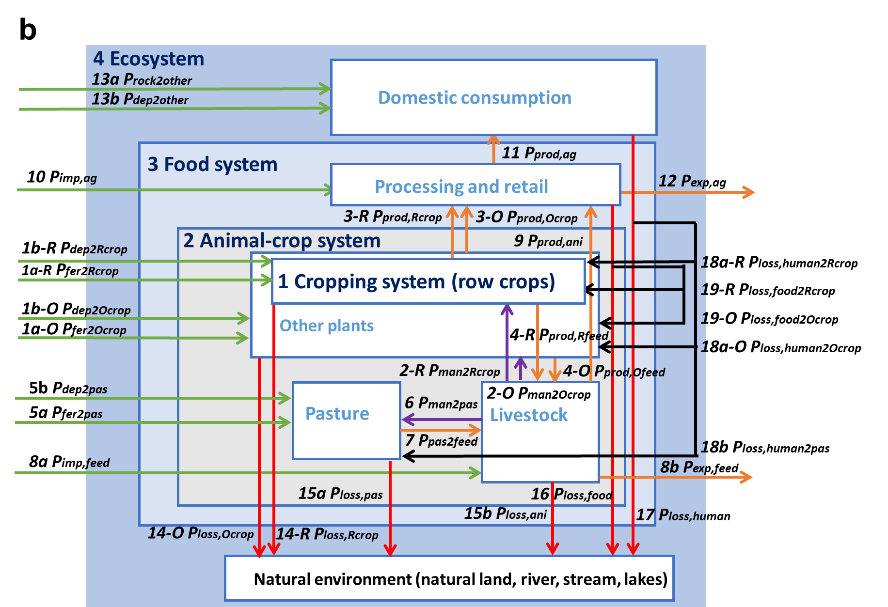


Figure S1. The nutrient budget database for N (a) and P (b). A detailed explanation of each system and flow can be found in Table S3 and Table S4. Green lines indicate external nutrient inputs; purple lines indicate recycled nutrient flows from livestock production; orange lines indicate productive nutrient outputs; red lines indicate nutrient loss to the environment (including air, soil, and water); black lines indicate nutrients recycled from food processing, retail, and consumption to soils. Dark blue texts indicate systems, and light blue texts indicate nutrient pools within system. R: row crops. O: other plants. Trade is represented by the net import and export flows.

# S2 The Chesapeake Bay watershed nutrient budget database

Most crop and animal production data are from the Chesapeake Assessment Scenario Tool (CAST-2019) database ^3^. Following the CAST-2019 database ^3^, the temporal scale of this study is from 1985 to 2019, with an annual record of 35 years. The spatial scale is the county scale in the Chesapeake Bay watershed. Some of these counties have portions outside the watershed boundary and they are all included in the analysis. There are 197 counties’ data of the seven jurisdictions (six states and DC) within the watershed recorded by the CAST-2019 database, which is the focus of this study. The study is on the county scale because this is the finest administrative unit with data in the CAST-2019 database. The counties/cities studied here, and their corresponding states/district, are listed in Table S1. The assessment on the watershed scale is conducted by aggregating the county level data.

Analyses were conducted for each county, with county boundaries shown in all figures. The location of the Chesapeake Bay (CB) watershed counties can be found in Figure S2o. County names can be found at <https://www.chesapeakebay.net/what/maps/chesapeake-bay-counties1>, and each county's Federal Information Processing System (FIPS) codes can be found in the supplementary database (see Data availability statement).

The first system is called the Cropping system, which focuses on row crop production. The second system is the Animal-crop system, which completes the agricultural production system and includes both crop and animal production. The third system is the Food system, which includes crop and animal production, and food processing and retail (including feed and food import). The last system, the landscape Ecosystem, includes the three previous systems and other nutrient-related human activities on the landscape, such as human food consumption and wastewater treatment. Most crop and animal production data are from the CAST-2019 ^3^. Nutrient flows not recorded by CAST-2019 (e.g., exported nutrients and nutrients consumed by humans) were estimated using assumptions and parameters (see S1 and S2 for more information).

## S2.1 Cropping system

Nutrient inputs to the Cropping system include mineral fertilizer, manure, deposition, fixation, and recycled waste (biosolids), as they are major inputs considered in nutrient studies ^4-9^ and they are quantified in CAST-2019.

CAST-2019 ^3^ provides various agricultural production data. Crop data were aggregated in CAST-2019 to 49 load sources, with each load source including one or more crop types. Load sources and crops included in the study are listed in Table S2. For the Cropping system, row crops are considered, which include ten load sources. Other agricultural plant and land types are considered in the Animal-crop system.

CAST-2019 data do not differentiate nutrients in feed crops for animals and crops for human consumption, so we assume a certain percentage of each type of crop will go to animal production or human consumption based on parameters in the literature. N and P yield by crop type in each load source was estimated using data kindly shared by Olivia Devereux from the Chesapeake Bay Program (CBP), then we multiplied the yield by the ratio of yield distributed for different purposes with parameters from the Net Anthropogenic Nitrogen Inputs (NANI) and Phosphorus Inputs (NAPI) Accounting Tool V3.1 ^10,11^. We assume that crops will be used for at least one of the five purposes after they are harvested: 1) consumed by humans as food, 2) consumed by humans as non-food, 3) exported, 4) consumed by animals as feed crops, and 5) others. Thus, the total nutrient in each nutrient flow for a specific purpose can be expressed as:

Equation S1

$${Removal}_{nu,cr,lo,co,yr,path}={Area}_{cr,lo,co,yr}\times{YieldPerArea}_{cr,lo,co,yr}\times{RemovalPerYieldUnit}_{nu,cr,lo}\times{Ratio}_{nu,cr,lo,path}$$

${Removal}_{nu,cr,co,yr,path}$ is the amount of nutrient type $nu$ (N or P) in crop type $cr$, load source $lo$, county $co$ and year $yr$ used for one of the five purposed $path$. ${Area}_{cr,lo,co,yr}$ is the area of crop type $cr$ under load source $lo$ in county $co$ and year $yr$ recorded by CAST-2019. ${YieldPerArea}_{cr,lo,co,yr}$ is the yield unit of crop type $cr$ per land area under load source $lo$ in county $co$ and year $yr$ recorded by CAST-2019. ${RemovalPerYieldUnit}_{nu,cr,lo}$ is the amount of harvested nutrient per yield unit of type $nu$ of crop type $cr$ and load source $lo$ recorded by CAST-2019. ${Ratio}_{nu,cr,lo,path}$ is the percentage of nutrient type $nu$ of crop type $cr$ under load source $lo$ used for human food, human non-food, export, feed, or others. A list of ${Ratio}_{nu,cr,lo,path}$ can be found in Table S5. The units were then converted to kg, km^2^, and hectare in the database to be consistent with other data. I then aggregated the crop level data to load source level data.


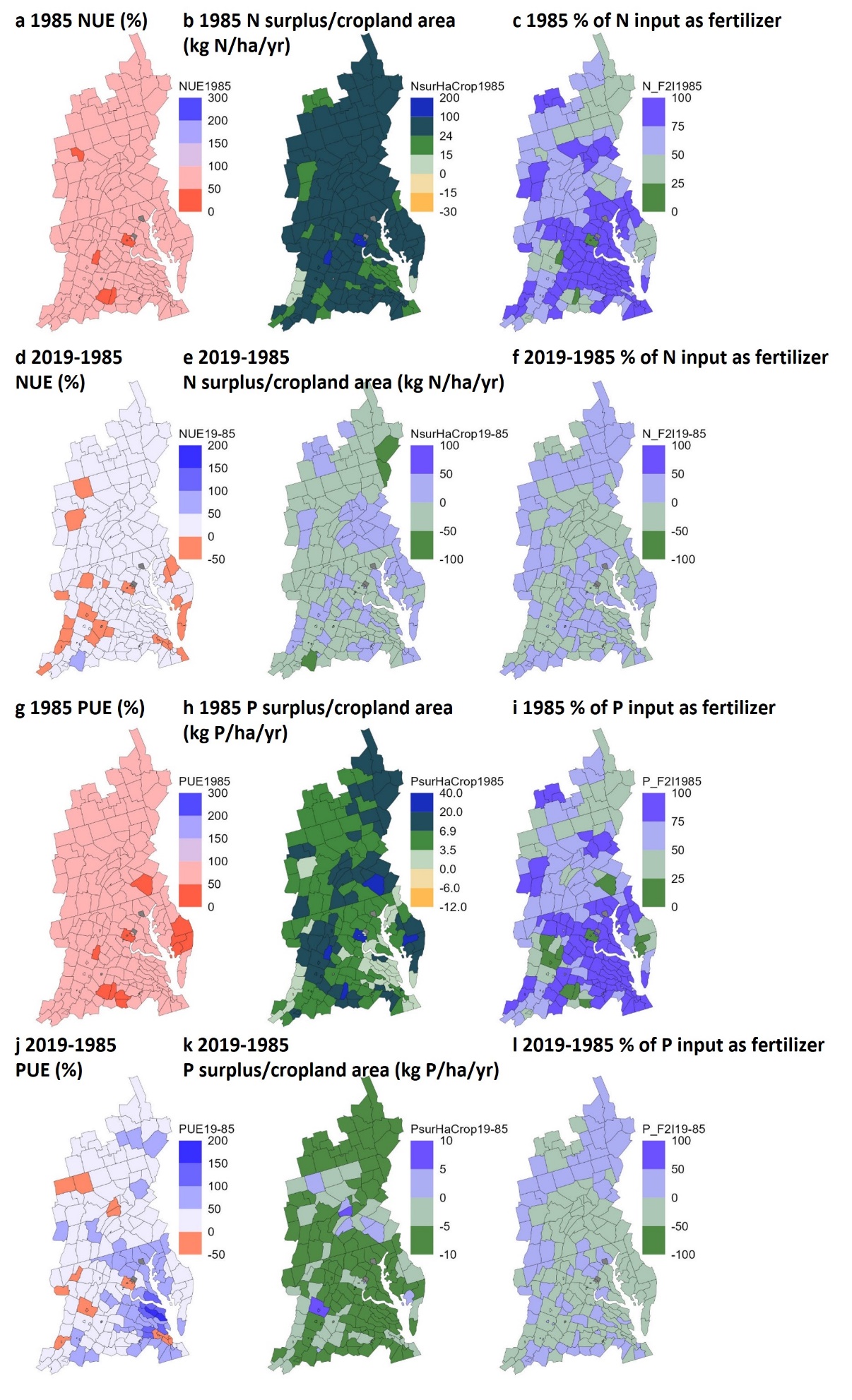


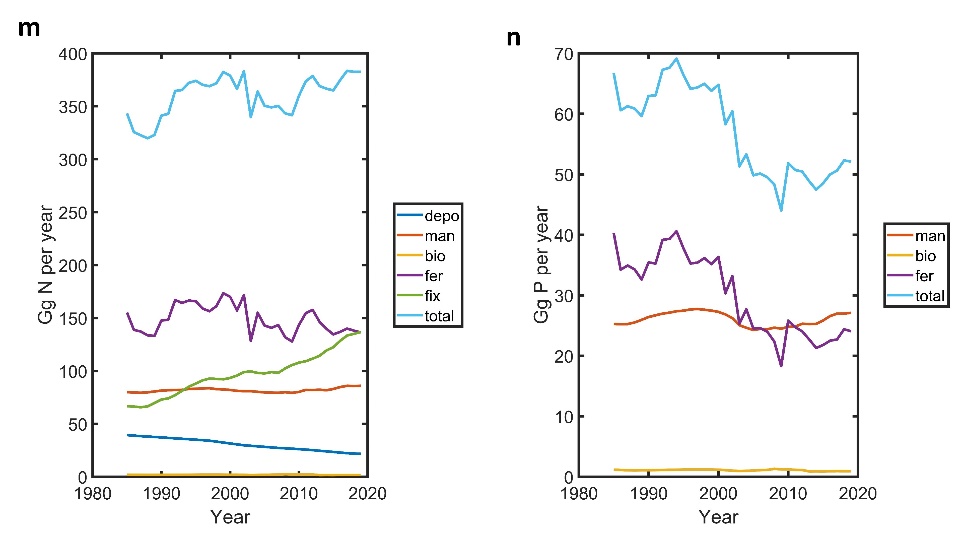


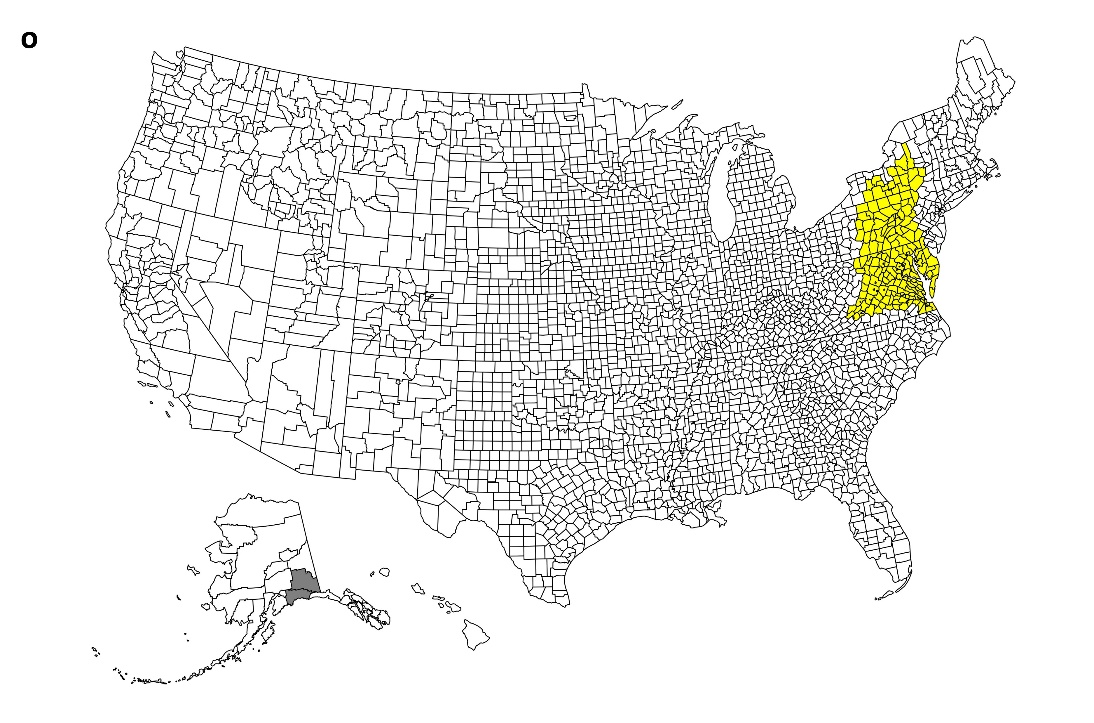


Figure S2. Cropland nutrient management performances in 1985 and changes between 1985 and 2019 (2019 values minus 1985 values) by county and nutrient input changes from 1985 to 2019 at the watershed scale. Cropping system NUE (a), PUE (g), NUE change (d), and PUE change (j), unit %. Cropland-based Cropping system N surplus (a), P surplus (h), N surplus change (e), and P surplus change (k), unit kgN or kg P per ha cropland area per year. Cropping system portion of the sum of mineral fertilizer and manure as mineral fertilizer for N (c), P (i), N change (f), and P change (l), unit %. Dark gray areas: no data. NUE and PUE values >100% represent potential soil mining. (m) Changes in Cropping system N inputs from 1985 to 2019 at the watershed scale. (n) Changes in Cropping system P inputs from 1985 to 2019 at the watershed scale. The inputs shown include "depo" (deposition input), "man" (manure input), "bio" (biosolid input), "fer" (mineral fertilizer input), "fix" (fixation input), and "total" (total input). (o) Location of the CB watershed counties in the US (watershed counties highlighted in yellow).

The soil plant-available P data estimated by year and county is organized by Sabo, et al. ^5^ (unit: kg P yr^-1^) and originally from the CAST-2019 database. We divide these data by county area (total land area by county) ^12^, to calculate the soil plant-available P distribution (kg P/km^2^ county area) in each county. According to CAST-2019, these data are estimated total soil plant-available P level in agricultural lands by county and year. The estimates were based on point data sampling and Mehlich 3, Bray, or Mehlich 1 soil plant-available P data. Methods varied by state and results were all converted to and reported in Mehlich 3. Because these soil test P measures are quick and easy, we can get a lot of measurements across space. They provide good indicators of P accumulation and broadly what should be available to crops now, but don't necessarily tell us about the breakdown of total P (e.g., will a gram of P potentially be available to a crop in 1 year, 3 years, 10 years, or only with a certain root system). Thus, there are uncertainties in the estimates. Data for P in other pools are currently not available and thus not discussed in this research. These uncertainties only affect the estimates of soil plant-available P but do not affect the estimates of other P budget data, such as PUE or P surplus. More details of soil data collection and calculation could be found in CAST-2019 model documentation ^3^.


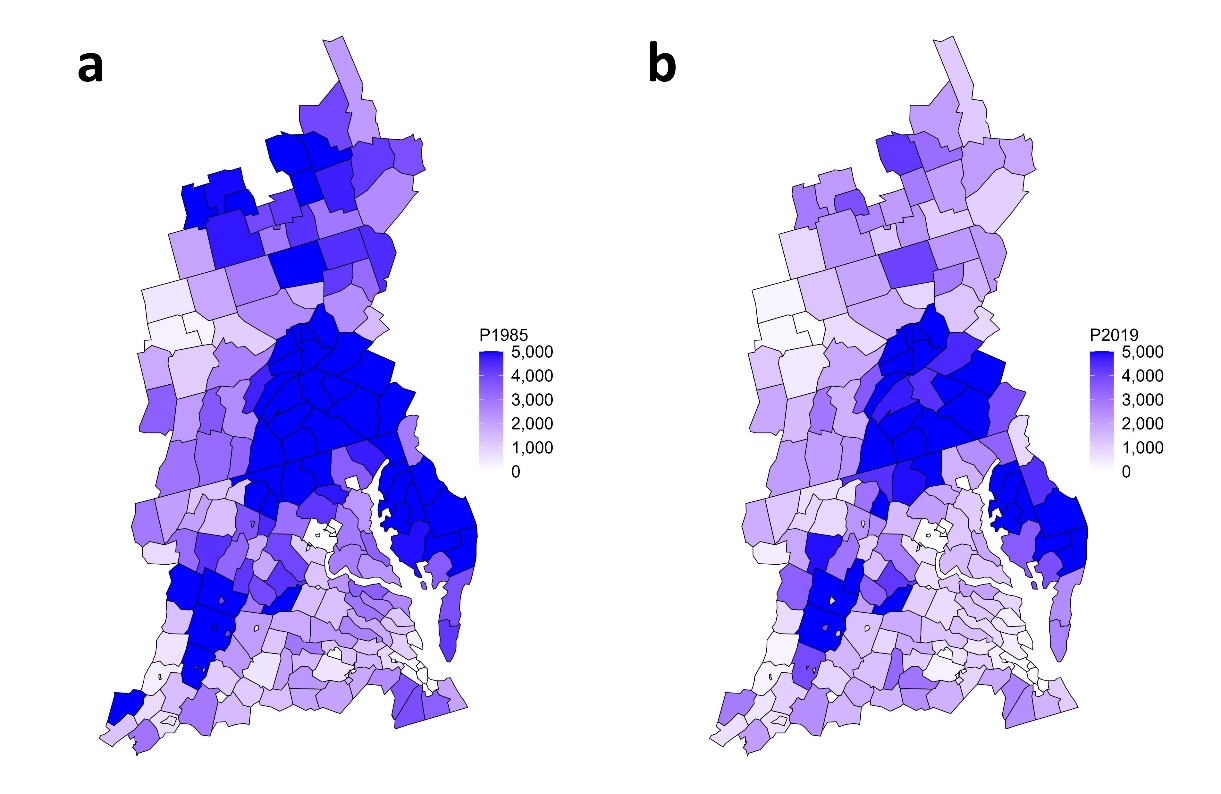


Figure S3**.** Mass of plant-available P in the soil (total annual agricultural soil plant-available P divided by county area in km^2^, unit kgP/km^2^) in 1985 and 2019. Gray areas are areas with no data. Values larger than 5,000 are colored as 5,000 for better visualization.

## S2.2 Animal-crop system

Animal-crop system lands include row crop lands in the Cropping system, riparian pasture, pasture, hay croplands, and agriculture open space. The application data of fertilizer and manure, atmospheric deposition, and fixation on different agricultural lands are from CAST-2019.

For animal production within the Animal-crop system, 12 major animal types that have production data in CAST-2019 were considered (Table S6). CAST-2019 ^3^ provides the number of animals in existence on an average day by animal type, year, and county, animal daily excretion rate, and nutrient content in manure they used to estimate animal nutrient budget, so N and P in directly excreted manure without any loss or further treatment can be estimated using their parameters and the methods mentioned in their document:

Equation S2

$${Excretion}_{nu,ani,co,yr}={AnimalNumber}_{ani,co,day}\times{DailyExcretionRate}_{ani,co,yr}\times365.25 days\times{DryFraction}_{ani}\times{NutrientConcentration}_{nu,ani,co,yr}$$

${Excretion}_{nu,ani,co,yr}$ is the annual total amount of nutrient $nu$ in manure excretion of animal type $ani$ in county $co$ and year $yr$. ${AnimalNumber}_{ani,co,day}$ is the number of animals on an average day of animal type $ani$ in county $co$ and year $yr$. ${DailyExcretionRate}_{ani,co,yr}$ is the daily excretion rate of animal type $ani$ in county $co$ and year $yr$. $365.25 days$ are average days in a year considering the leap year. ${DryFraction}_{ani}$ is the dry fraction of manure of animal type $ani$. ${NutrientConcentration}_{nu,ani,co,yr}$ is the concentration of total nutrient $nu$ per unit weight of dry manure of animal type $ani$ in county $co$ and year $yr$. All variables are downloaded from CAST-2019 ^3^. The excreted manure could be left on pasture, lost to the environment, applied to soils, or transported to other counties.

After nutrients are consumed by animals, part of it will be excreted (estimated above), and the rest will be stored in animals (called nutrient production). The nutrients stored in animals can be divided into two parts, including edible portion (defined as the portion transported to and consumed by humans) and inedible portion (defined as the portion discarded or used for non-human purposes such as pet food). The focus of this study is on human consumption, as data on the consumption of pets and other animals by county and year is currently unavailable. Figure S4 illustrates where the nutrients will go after they are consumed by animals in this model.


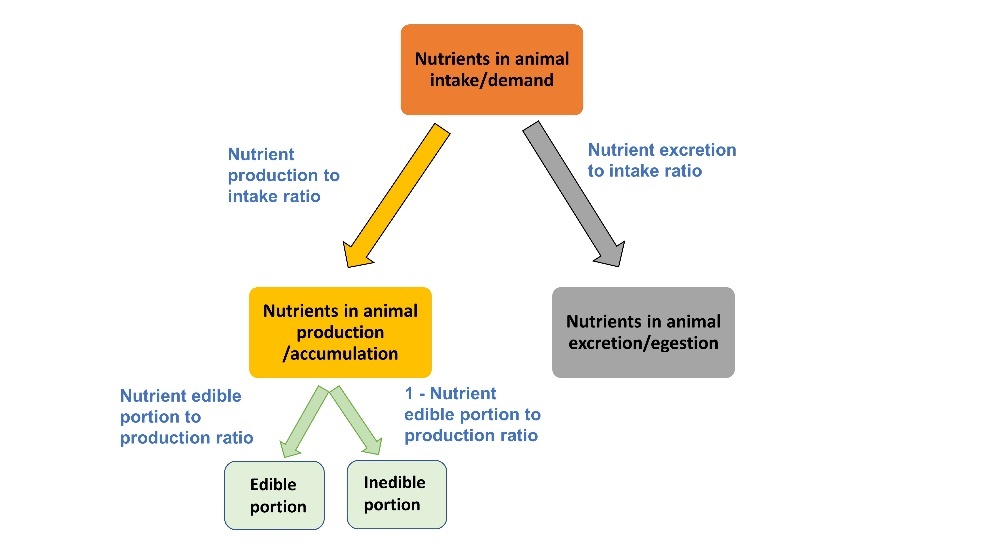


Figure S4. Illustration of nutrient fate in animals (arrows and boxes) and parameters used to estimate nutrient flows (texts by the sides of arrows) in animal production.

We then applied the nutrient consumption, excretion, and production parameters in the literature to estimate nutrient excretion to nutrient intake ratio and the edible portion to nutrient production ratio (Table S6). With these ratios we calculated nutrient consumption and production and the excretion estimated. For the edible portion, following the definition in a previous study ^13^, only boneless meat, half of the offal, and half of the fat from the carcass were considered. We further assume that the edible portion in nutrient production is 0% for horses, considering that horses are traditionally not for food in the US ^14^. For milk cows, we assume that all nutrients consumed, after part of them go to excretion, are converted to milk, following the assumption of a previous study ^15^. As more detailed data becomes available on the allocation of nutrients to different pathways, these estimates will be updated to provide a more accurate representation of the distribution of nutrients. The productive outputs of the Animal-crop system include both the edible and inedible portions, which will be later separated in the Food system after food processing and retail.

Animal-crop production may include the import and export (trade) of feed crops. Due to data unavailability, we assume that the import of feed crops only happens if local production cannot meet local demand, and the export of feed crops will happen if local production is larger than the estimated local demand (animal consumption estimated above). The net import of nutrients in feed crops was estimated as:

Equation S3

$${FeedNetImport}_{nu,co,yr}= \sum_{ani} {FeedConsumption}_{nu,ani,co,yr}-\sum_{cr} \left( 1-{Ratio}_{loss,nu,cr} \right)\times{FeedCrops}_{nu,co,cr,yr}-{pasture}_{nu,co,yr}$$

${FeedNetImport}_{nu,co,yr}$ is the total amount of net feed import (excluding import loss) nutrient of nutrient $nu$ in county $co$ and year $yr$. ${FeedConsumption}_{nu,ani,co,yr}$ is the nutrients consumed by animals of nutrient $nu$ in county $co$ and year $yr$ by animal type $ani$ estimated above. ${Ratio}_{loss,nu,cr}$ is the loss ratio of nutrients in feed crops of nutrient $nu$ and crop type $cr$. ${FeedCrops}_{nu,co,cr,yr}$ is the nutrient amount in feed crop $cr$ of nutrient $nu$ produced in county $co$ and year $yr$ estimated in the Cropping system section. ${pasture}_{nu,co,yr}$ is the amount of nutrient $nu$ harvested from pasture in county $co$ and year $yr$ available from CAST-2019. I assume ${Ratio}_{loss,nu,cr}$ is 20% for hay and silage and 10% for other crops ^16^.

If the net import is positive, it is considered as an import and assume the export is 0. If the net import is negative, it is considered as export and assume import is 0. If the net import is zero, both import and export are assumed 0.

For the Animal-crop system, the influence of animal production on the system budget was also investigated. The indicator of broiler density was used to represent animal production, which is defined as the number of broilers per county per year divided by the county land area. Broiler production is an important contributor to nutrient loads in the Bay watershed. For example, the averaged data from 2015-2019 suggest that broilers contributed approximately 25% of the total N and P in manure in the Bay watershed, 51% of total N in animal products, and 32% of total P in animal products, which is larger than the contribution of any other type of animal production.


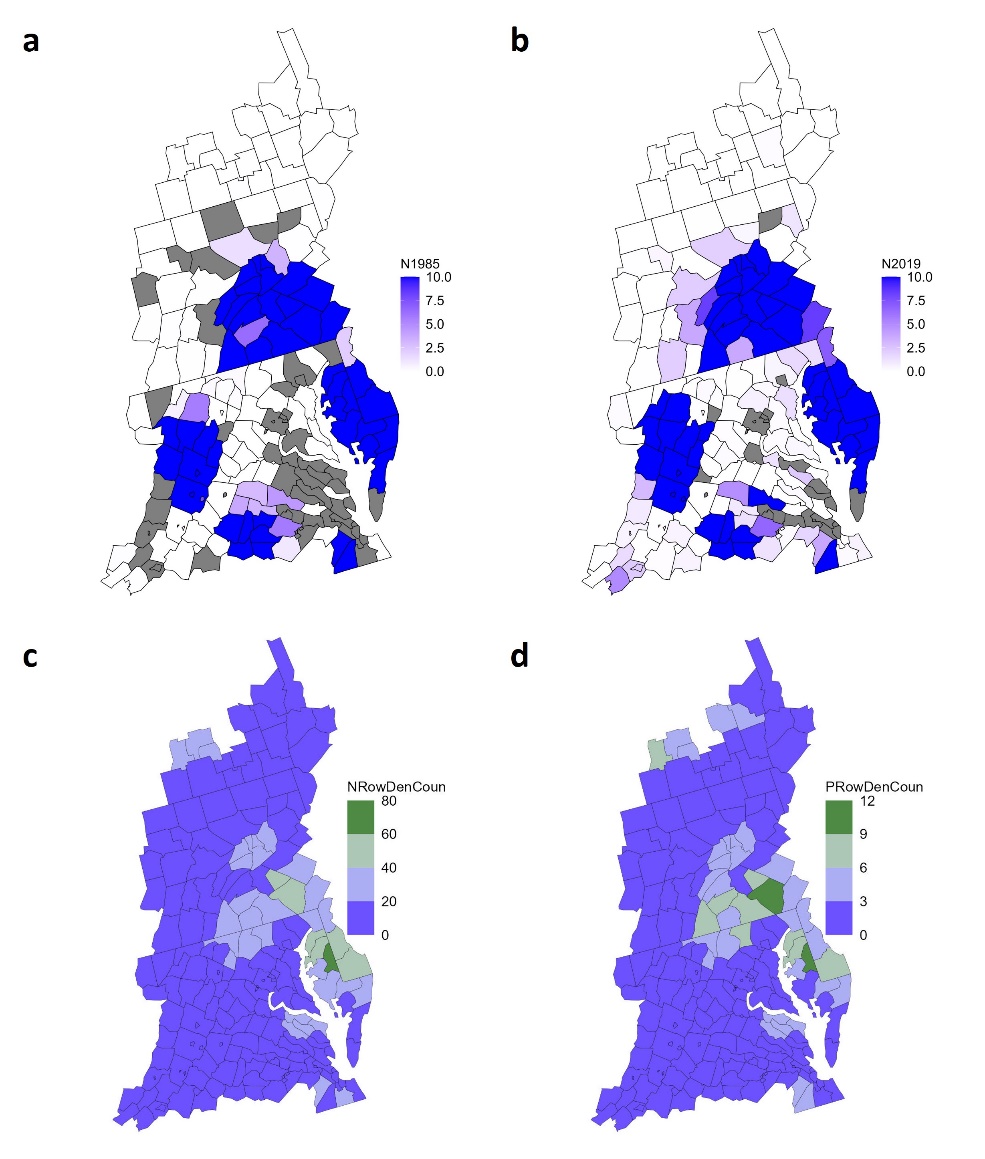


Figure S5**.** Broiler and crop production density by county. Broiler density by county (count/county area in ha) in 1985 (a) and 2019 (b). Cropping system N production density (N crop production in kg N/county area in ha, panel c) and P production density (P crop production in kg P/county area in ha, panel d) in 2019. Grey areas are areas with no data. Values larger than 1,000 are colored as 1,000 for better visualization.

## S2.3 Food system

The food system receives imported feed and food products, and its productive outputs include nutrients in exported non-food crops estimated in the Cropping system section, exported animal products estimated in the Animal-crop system section, exported food products, food consumed locally, and non-food products consumed locally.

Food consumption is estimated as the product of county population and food consumption parameter, which is 6.21 kg N per person per year based on U.S. statistics on daily protein consumption ^10,17^ and 0.64 kg P per person per year ^4,10,18^. Due to data limitation, we simply assume all cotton and tobacco (belonging to the “exported food products” group) will be exported following the previous studies ^10,11^, and non-food agricultural products (excluding crops belonging to the “exported food products” group) produced locally are consumed locally. Following the methods used to estimate imported and exported feed crops in the last section, the net import of food products was estimated as the difference between local production and local demand:

Equation S4

$${FoodNetImport}_{nu,co,yr}= {FoodConsumption}_{nu}\times{Population}_{co,yr}-\sum_{ani} \left( 1-{Ratio}_{loss,nu,ani} \right)\times{AnimalProduct}_{nu,co,ani,yr}-\sum_{cr} \left( 1-{Ratio}_{loss,nu,cr} \right)\times{FoodCrops}_{nu,co,cr,yr}$$

${FoodNetImport}_{nu,co,yr}$ is the total amount of net food import nutrient of nutrient $nu$ in county $co$ and year $yr$. ${FoodConsumption}_{nu}$ is the food nutrients consumed by people of nutrient $nu$ per year, and ${Population}_{co,yr}$ is the population in county $co$ and year $yr$ ^19^. ${Ratio}_{loss,nu,ani}$ is the loss ratio of nutrients in animal products of nutrient $nu$ and animal type $ani$. ${AnimalProduct}_{nu,co,ani,yr}$ is the nutrient amount in animal products (edible portion) of animal $ani$ of nutrient $nu$ in county $co$ and year $yr$ estimated in the Animal-crop system section. ${Ratio}_{loss,nu,cr}$ is the loss ratio of nutrients in food products of nutrient $nu$ and crop type $cr$. ${FoodCrops}_{nu,co,cr,yr}$ is the nutrient amount in food products of crop $cr$ of nutrient $nu$ in county $co$ and year $yr$ estimated in the Cropping system section. ${Ratio}_{loss,nu,ani}$ and ${Ratio}_{loss,nu,cr}$ is assumed at 10% ^16^.

If the net import is positive, it is assumed to be import, and export is 0. If the net import is negative, it is assumed to be export and import is 0.


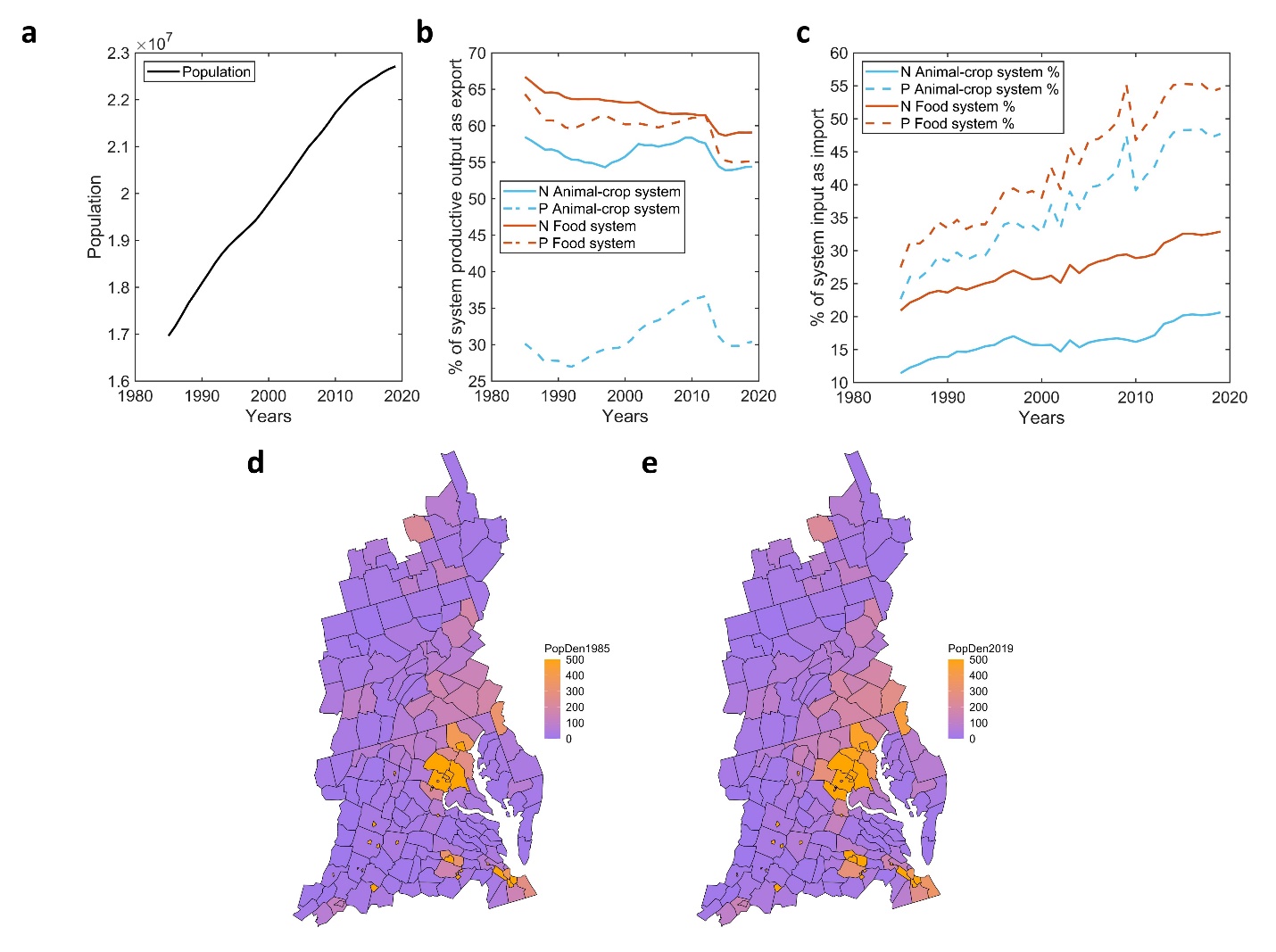


Figure S6. Watershed population and trade change. (a) Population annual change on the watershed level. (b) Percentage of system productive output as net exported nutrients (from each county to other regions insides or outside the CB watershed) by year, system, and nutrient type (c) Percentage of system input as net imported nutrients (to each county from other regions insides or outside the CB watershed) by year, system, and nutrient type. For import and export, Cropping system is not plotted since trade is not included in this system, and Ecosystem is also not plotted since export is 100% of productive output in this system (Figure S1). Note that the sum of net import or net export at the watershed level is the sum of all counties’ net import or export, not the import or export between CB watershed and non-watershed regions. (d) Population density (people/county area in km^2^) by county in 1985. (e) Population density (people/county area in km^2^) by county in 2019.

## S2.4 Ecosystem

The productive outputs of the Ecosystem are agricultural products (food and non-food) exported from this region, estimated in the last section. CAST-2019 provides urban fertilizer use for N and P. P in detergents was included in the inputs and the parameters used by state and year can be found in Table S7 and Table S8.

The amount of nutrients in wastewater was also quantified to estimate how much N and P can be recycled. The total point source loads estimated here are the sum of municipal wastewater treatment loads, industrial wastewater treatment loads, combined sewage overflows, and septic downloaded from CAST-2019 ^5^.

## S2.5 Discussing both nutrient use efficiency and nutrient surplus

Both nutrient use efficiencies and potential nutrient losses are quantified and discussed, because the magnitude of potential nutrient loss is not always directly associated with nutrient use efficiency. For example, a region may have a highly efficient system but also a large nutrient surplus due to high inputs ^20^.

Although nutrient use efficiency and nutrient surplus correspond to each other (i.e., nutrient use efficiencies over 100%, at 100%, and below 100% indicate negative, zero, and positive nutrient surplus, respectively), it is still useful to discuss both nutrient use efficiency and nutrient surplus. Nutrient use efficiency and the sign (i.e., positive or negative) of nutrient surplus indicate how efficiently nutrients are used, but they do not reveal the magnitude of potential nutrient losses. For example, a region with a relatively high nutrient use efficiency but significantly larger nutrient inputs might experience greater nutrient losses and a more substantial environmental impact, compared to a place with lower nutrient use efficiency but much smaller nutrient inputs.

## S2.6 Nutrient use efficiency and surplus changes across the time

As expected when surpluses increase, both NUE and PUE decreased from the Cropping system to Ecosystem in 1985 and 2019 (Figure 4). This was mainly due to a larger increase in system inputs and a smaller increase or even decrease in system productive outputs from Cropping system to higher-level systems for both N and P, and an increase in N system inputs from 1985 to 2019 (Table 1).

Our results show that at least half of the counties had NUE and PUE in systems beyond croplands lower than Cropping system efficiency in 1985 and 2019. For the Ecosystem in 2019, more than 90% of counties had an NUE or PUE smaller than the Cropping system efficiency.

These temporal changes in nutrient use efficiencies and surpluses are the long-term results of mixed factors, including changes in nutrient input and output flows over time, as well as the long-term efforts and effects of nutrient management policy and strategy in the CB watershed.

By comparing different nutrient flows from 1985 to 2019 in detail (Figure 2 and Figure S2 m-n), we can observe the following:

1. For N, there was an increase in NUE and a decrease in N surplus between 1985 and 2009, but a slight decrease in NUE and an increase in N surplus during 2009-2019. While the improvement during 1985-2009 can be attributed to long-term advancements in nutrient management since 1984 and even earlier, the transition since 2009 can be explained by increased N in harvested crops and much larger increases in N inputs. Comparing different N input flows, although the changes in N mineral fertilizer and manure inputs from 2009 to 2019 were relatively stable with a slight increase, and the N deposition input even decreased slightly, the significantly increased N fixation is the major reason for the obviously increased N inputs. The increased N fixation is correlated with the increased N in the harvested crops.

2. For P, the significant increase in PUE and decrease in P surplus between 1985-2009 can be attributed to improvements led by long-term policy and strategy changes and effects (see the paragraph below). During 2009-2019, the decreasing PUE and increasing P surplus can mainly be explained by the stable P amount in harvested crops, slightly increased manure P input, and increased P mineral fertilizer input since 2009.

To control nutrient pollution, a series of agreements were signed by the U.S. Environmental Protection Agency (USEPA), CB watershed states, and the Chesapeake Bay Commission (CBC). The first Chesapeake Bay Agreement, signed in 1984, included four states (Maryland, Pennsylvania, Virginia, and the District of Columbia) and emphasized the necessity of cooperative action to clean up the Bay ^21,22^. The second Agreement, signed in 1987, set a goal of a 40% nutrient pollution reduction by 2000. The third Agreement, signed in 1992, reaffirmed the previous commitments. In 2000, the fourth Agreement was signed, including three additional states (Delaware, New York, and West Virginia) and set a cleanup deadline of 2010 ^22^. In 2010, the USEPA developed the Chesapeake Bay Total Maximum Daily Load (Bay TMDL), specifying limits on nitrogen, phosphorus, and sediment pollution entering the Bay and tidal rivers. Each of the seven Bay jurisdictions created their own cleanup plans to meet their pollution reduction goals by 2025 in Phase I Watershed Implementation Plans (WIPs), forming the Chesapeake Clean Water Blueprint ^22,23^. There have also been state-level policy changes since 2000, including the Nutrient Management Law passed in the early 2000s in Maryland ^5^, the first Nutrient Management Law in the nation passed in Pennsylvania in 1993 and reviewed in 2005 ^24^, the adoption of the P Site Index as a BMP in Delaware ^25^, the fourth Chesapeake Bay Agreement signed in 2000 ^22^, and the Chesapeake Bay Tributary Strategies developed by the seven watershed jurisdictions in 2000s ^26^.

## S2.7 Strategies to improve nutrient management in each system

To improve nutrient management in each system, a range of strategies can be implemented: 1) at crop production: improving cropland fertilization efficiency, optimizing N:P ratios in agricultural inputs, applying cost-effective technologies to use legacy P and N in the soil and water, and reducing the loss of dissolved P ^27,28^; 2) at animal production: improving animal diets to increase efficiency of nutrient utilization by animals and reduce nutrient excretion and emissions, increasing nutrient recovery in manure, optimizing livestock production location and density to facilitate nutrient management and recycling, and reducing nutrient runoff from areas with high animal densities ^16,27,29^; 3) at food processing and retail stages: increasing nutrient use monitoring and improving data availability, improving nutrient recovery from nonedible food and wasted edible food, optimizing food redistribution to reduce nutrient loss ^30,31^; 4) at human food consumption and waste management stages: promoting healthy diets with smaller nutrient footprints, continuing to upgrade wastewater treatment facilities to increse the recyling of nutrients from human waste and feasibly extracting nutrients for agricultural and horticultural production, reducing nutrient losses in stormwater runoff, reducing nutrient losses from urban lawns, encouraging the composting of food waste and reducing the volume of food waste sent to landfills ^32-37^.

# S3 Theoretically recyclable waste

An illustration of the nutrient flows discussed above can be found in Figure S7.


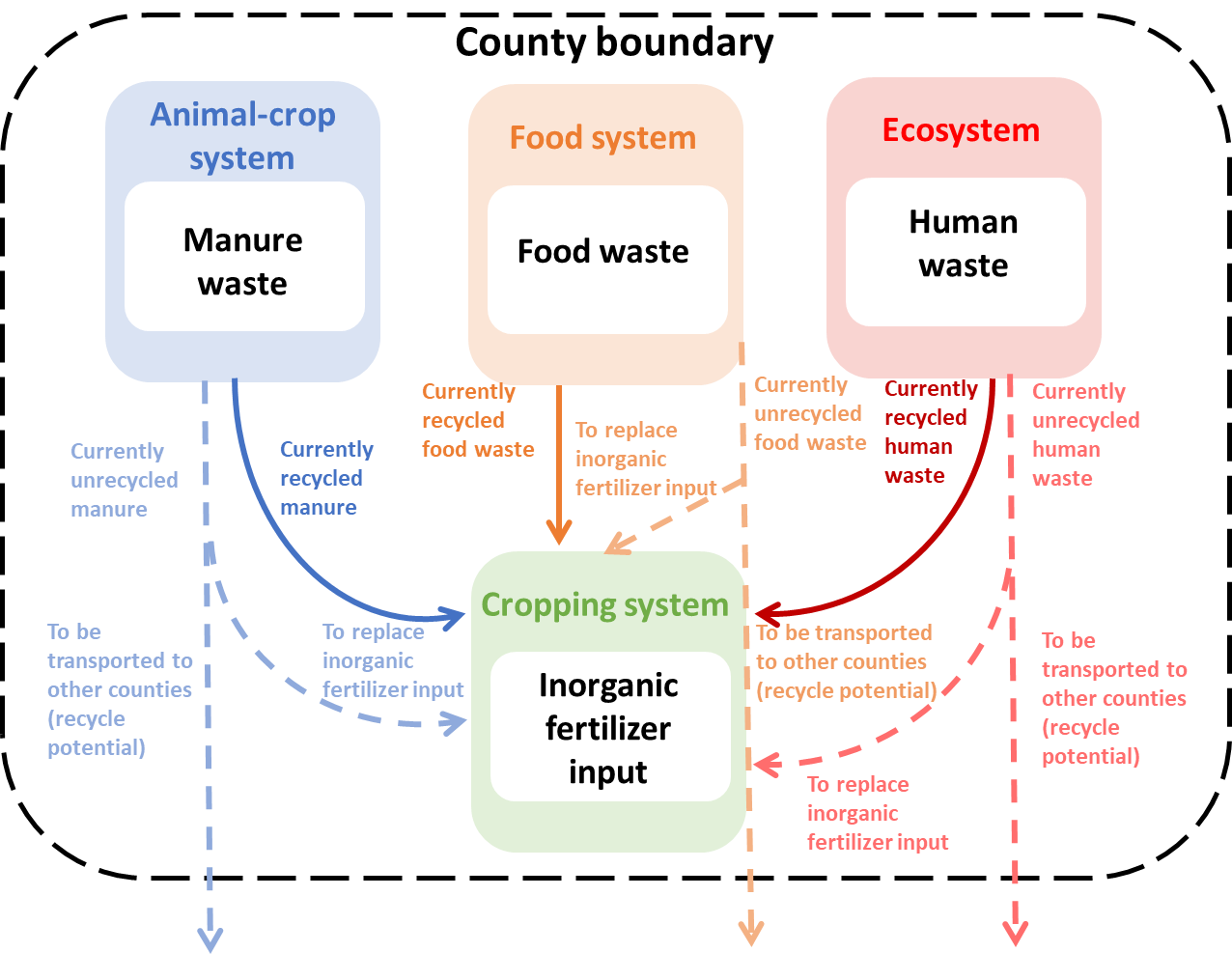


Figure S7. An illustration of recycled nutrients and unrecycled (theoretically recyclable) nutrients from manure, food waste, and human waste. Soil line: currently recycled waste. Dashed line: currently unrecycled waste.

Theoretically recyclable manure defined in this study is the difference between the total amount of manure excreted locally and the amount of manure that has already been recycled locally. We defined recycled nutrients in manure as those that have been used locally including both the manure currently applied to soils by humans for agricultural production, as well as the manure directly deposited on the soil by animals. The theoretically recyclable nutrients in manure are the parts used for other purposes (e.g., energy) or lost to the environment during collection, storage, and transportation.

The theoretically recyclable food waste analyzed in this study is defined as the waste generated from food processing and retail operations, including 10% of food crop products and 10% of the edible portion of animal products, and the inedible portion of animal products estimated in the Food system.

Theoretically recyclable human waste includes total nutrients in municipal and industrial wastewater treatment loads, combined sewage overflows, and septic.


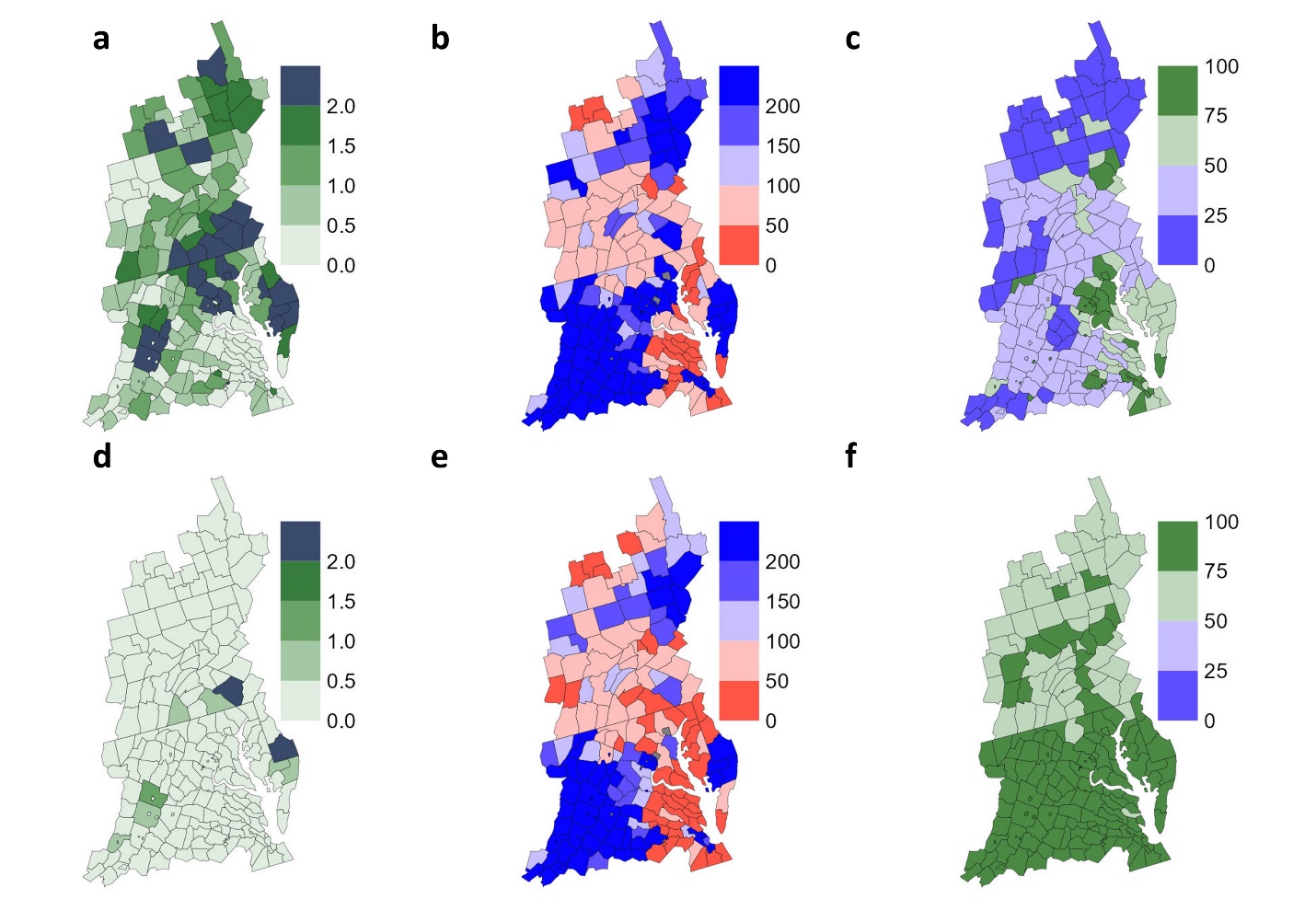


Figure S8**. Theoretically recyclable nutrients by county in 1985.** Total theoretically recyclable N and P (a and d, unit Gg N or Gg P/yr). The percentage of local fertilizer demand that can be offset by total theoretically recyclable waste (b and e, unit %). The percentage of total theoretically recyclable N and P from human and food waste (versus unrecycled manure, panel c and f, unit %).

Technical, logistical, market, and environmental challenges for nutrient waste recycling include: 1) effectively tracking, collecting, and extracting nutrients from waste; 2) transporting waste; 3) reducing processing and transporting cost and distance; 4) reallocating nutrients to different regions by matching the N:P ratio and nutrient amount in waste with that required by croplands ^35,38,39^; 5) addressing environmental and health concerns associated with food waste and human waste (e.g., odors, pathogens, heavy metals, organic and PFAS (per- and polyfluoroalkyl substances) contamination, and legal requirements) ^27,38,40-43^; 6) achieving the same agronomic benefits as those obtained through the application of mineral fertilizer. For example, Margenot et al. ^44^ argue that achieving full manure recycling may not be agronomically viable to meet the crop's P demands. Addressing those socioeconomic and technical barriers to increasing waste recycling would require further study and considerable investment in policy, technology, and logistics.

Efforts have been made to overcome these challenges and barriers. For instance, the U.S. Department of Agriculture and the USEPA have announced the U.S. 2030 Food Loss and Waste Reduction goal, which aims to halve food loss and waste by 2030 compared to the baseline year of 2016 ^45^. Additionally, there are emerging treatment technologies to move PFAS from wastewater ^46^, and the USEPA has released methods to more accurately measure PFAS and proposed PFAS-related rules under the Resource Conservation and Recovery Act in early 2024 ^47^. Also, the concept of “manuresheds” has been proposed to link counties with surplus manure nutrients to those with nutrient demands, taking into account factors such as the N:P ratio in manure, the distance between counties, the availability of manure nutrients, and the demand for nutrients ^38^.

# S4 Statistical analysis

To compare the medians of more than two groups of data and to see whether they are statistically different, the Kruskal-Wallis test was applied ^48^. This is a nonparametric test, which does not require a normal distribution of data. Linear regression was also applied to study the relationship between different variables.


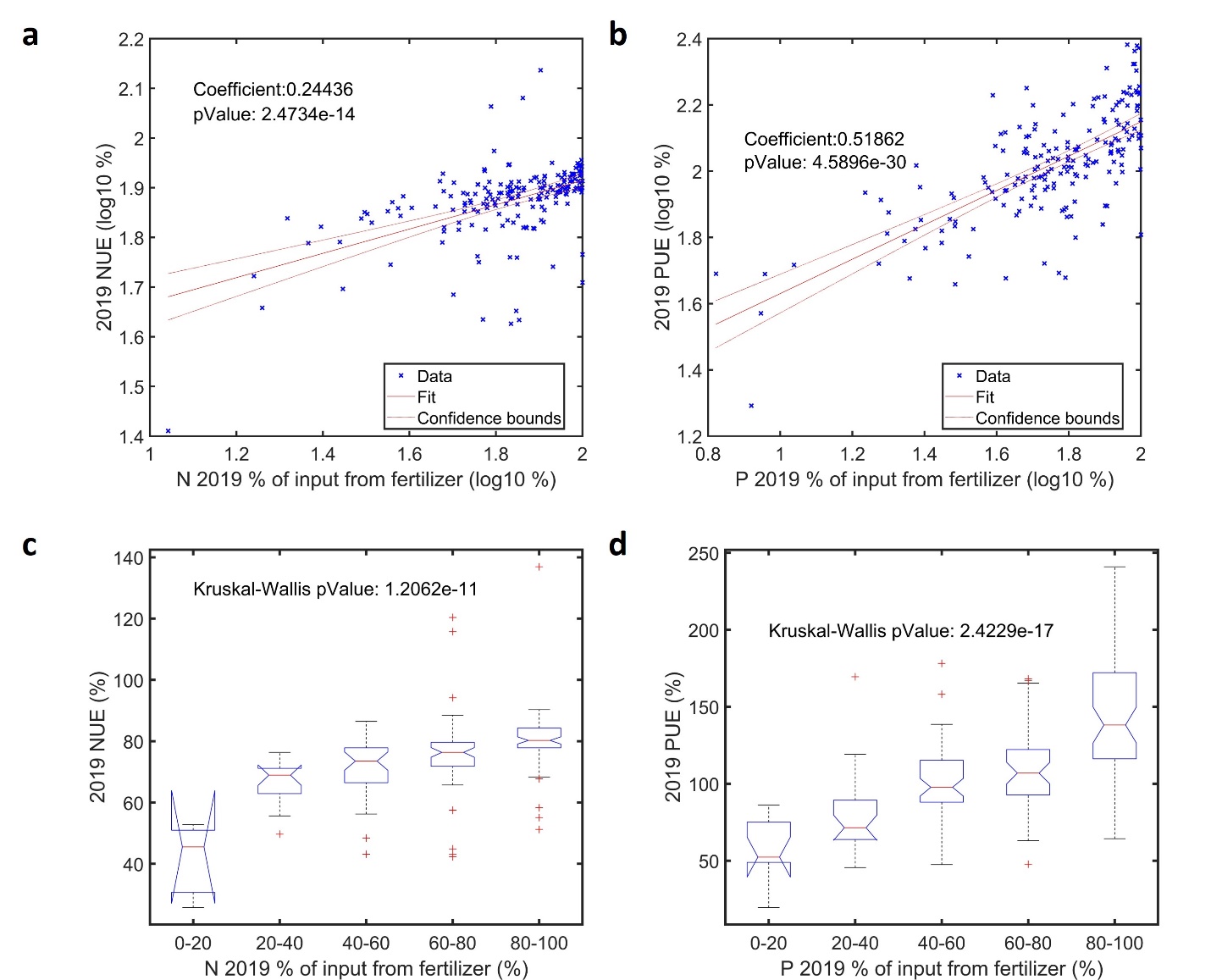


Figure S9**.** Regression results and Kruskal-Wallis test results for NUE, PUE, and % of input as fertilizer in 2019. For linear regression, log transformation was applied to data. Box plots: displaying the minimum, 25^th^ percentile, median, 75^th^ percentile, and maximum values, and outliers of county-level data.


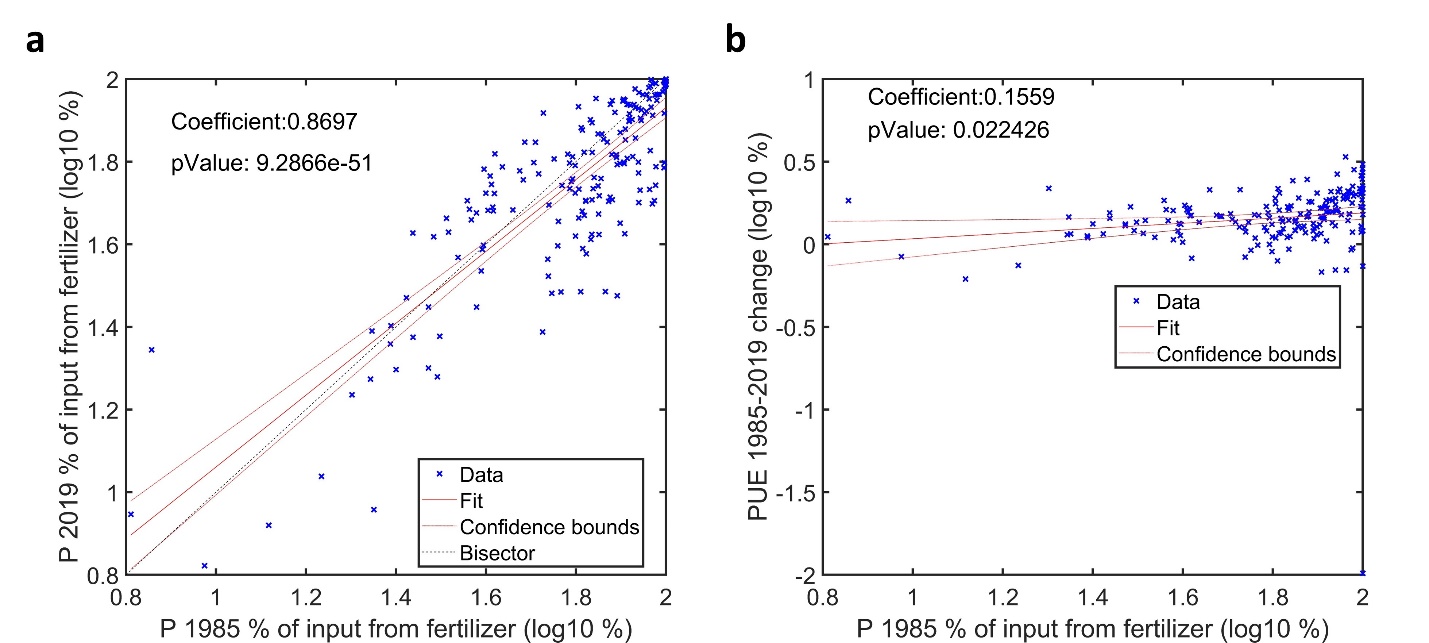


Figure S10. Temporal change of P % of input as fertilizer and PUE. (a) P % of input as fertilizer in 1985 and in 2019. (b) PUE change during 1985 and 2019 and P % of input as fertilizer in 1985. All data are in log form.


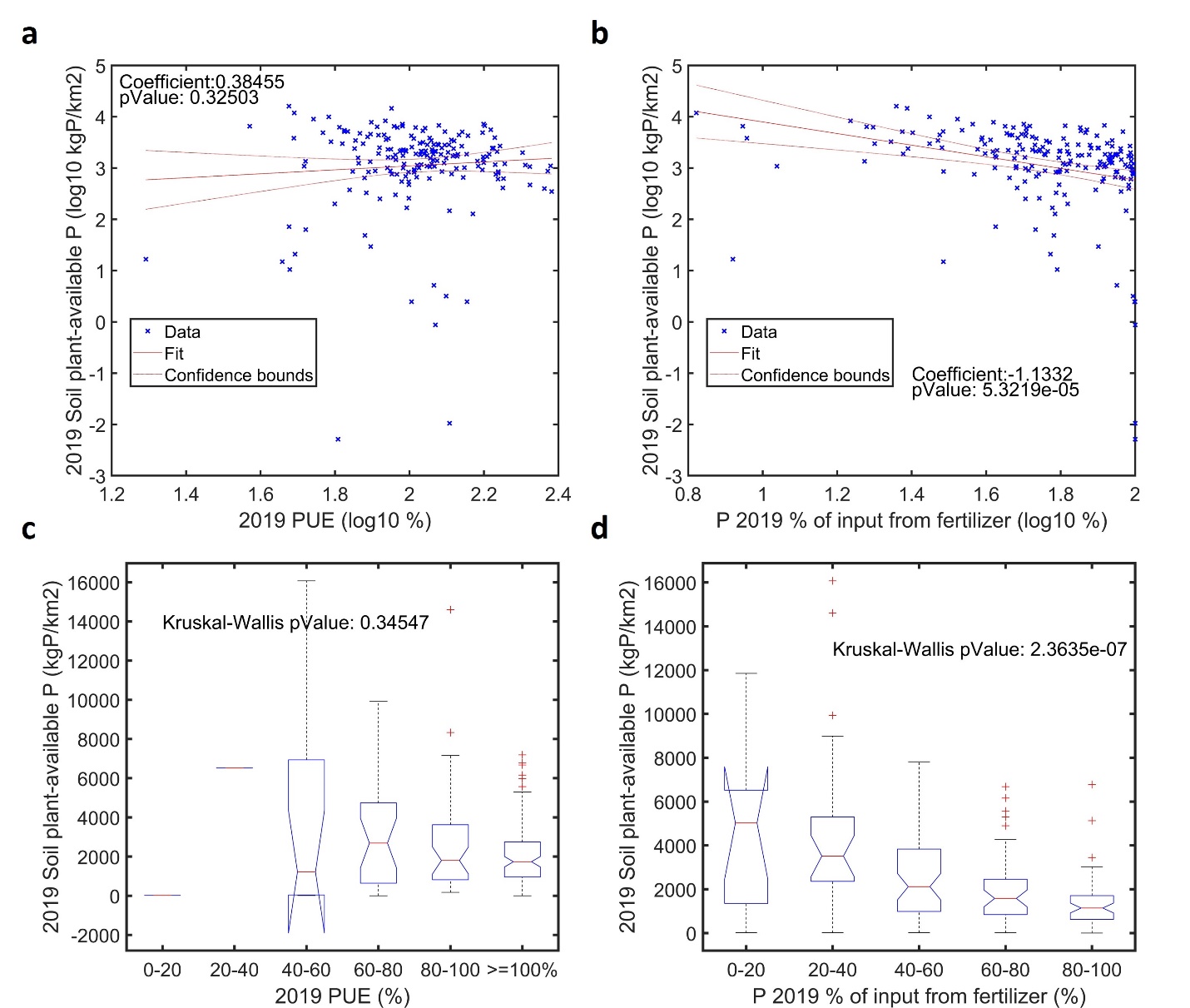


Figure S11**.** Regression results (a,b) and Kruskal-Wallis test results (c,d) for Soil plant-available P, PUE, and P % of input from fertilizer in 2019. For linear regression, log transformation was applied to data. Box plots: displaying the minimum, 25^th^ percentile, median, 75^th^ percentile, and maximum values, and outliers of county-level data.


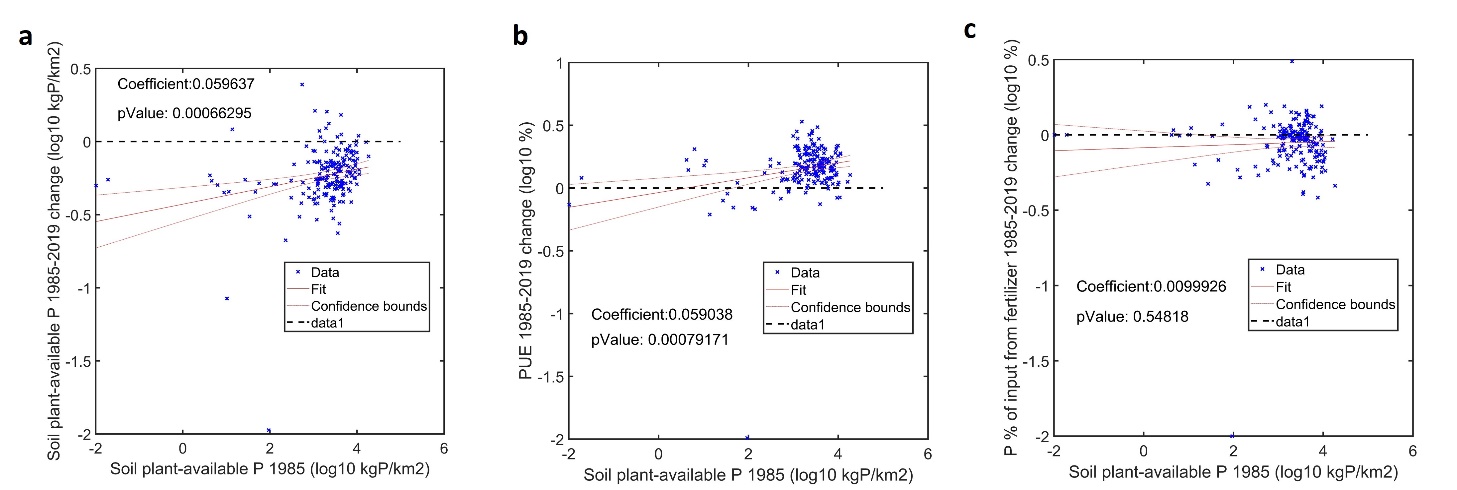


Figure S12. Comparing the temporal changes of soil plant-available P, PUE, and P % of input from fertilizer. All data are in log form. (a) 1985 soil plant-available P versus soil plant-available P 1985-2019 temporal change. (b) 1985 soil plant-available P versus PUE 1985-2019 temporal change. (c) 1985 soil plant-available P versus P % of input from fertilizer 1985-2019 temporal change. Each point represents one county data. The horizontal line in each plot indicates whether a county experienced an increase or decrease in y axis variable value from 1985 to 2019.

# S5 Nitrogen to phosphorus ratio in surplus

The N to P ratio in surplus is defined as the ratio of N surplus to P surplus in each system. Negative surpluses are treated as zero since here we focus on positive nutrient loss potential only, and it is hard to interpret negative ratios. Infinite values are not included in the analysis. A ratio larger than 1 indicates a larger loss potential for N than that of P, and ratio lower than 1 indicates that P has a larger loss potential than N.

Equation S5

$$N to P ratio in surplus=\frac{system N surplus}{system P surplus}$$

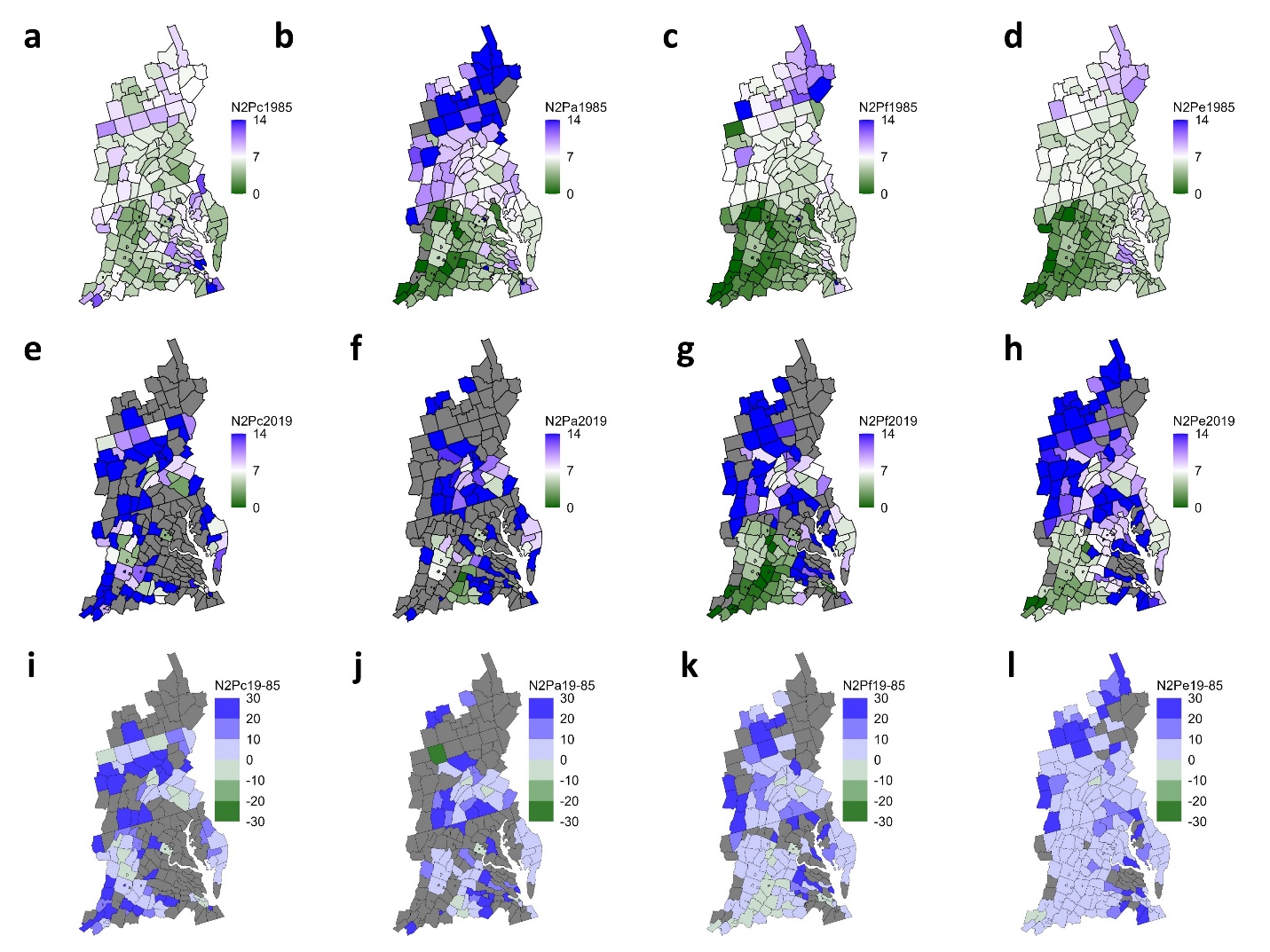


Figure S13. N to P ratios in surpluses by county across systems in 1985 and 2019. (a-d) N:P ratios in surpluses by county across systems in 1985. (e-h) N:P ratios in surpluses by county across systems in 2019. (i-l) changes of N:P ratios in surpluses by county across systems (2019 value minus 1985 value). “N2P”: N surplus to P surplus ratio. “c”: Cropping system. “a”: Animal-crop system. “f”: Food system. “e”: Ecosystem. Gray areas are areas with no data. Negative surpluses and infinite ratios are not included for analysis, causing missing data in the maps. Values larger than 14 are colored as 14 for better visualization.

# S6 Definition and parameters

Table S1. The complete list of counties/cities and their corresponding states/district considered in this study ^3,6^.

| State/District | State/District abbreviation | Counties/Cities total number | Counties/Cities |
| --- | --- | --- | --- |
| District of Columbia | DC | 1 | District of Columbia |
| Delaware | DE | 3 | Kent, New Castle, Sussex |
| Maryland | MD | 24 | Allegany, Anne Arundel, Baltimore, Calvert, Caroline, Carroll, Cecil, Charles, Dorchester, Frederick, Garrett, Harford, Howard, Kent, Montgomery, Prince Georges, Queen Annes, St. Marys, Somerset, Talbot, Washington, Wicomico, Worcester, Baltimore City |
| New York | NY | 19 | Allegany, Broome, Chemung, Chenango, Cortland, Delaware, Herkimer, Livingston, Madison, Oneida, Onondaga, Ontario, Otsego, Schoharie, Schuyler, Steuben, Tioga, Tompkins, Yates |
| Pennsylvania | PA | 43 | Adams, Bedford, Berks, Blair, Bradford, Cambria, Cameron, Carbon, Centre, Chester, Clearfield, Clinton, Columbia, Cumberland, Dauphin, Elk, Franklin, Fulton, Huntingdon, Indiana, Jefferson, Juniata, Lackawanna, Lancaster, Lebanon, Luzerne, Lycoming, Mckean, Mifflin, Montour, Northumberland, Perry, Potter, Schuylkill, Snyder, Somerset, Sullivan, Susquehanna, Tioga, Union, Wayne, Wyoming, York |
| Virginia | VA | 96 | Accomack, Albemarle, Alleghany, Amelia, Amherst, Appomattox, Arlington, Augusta, Bath, Bedford, Botetourt, Buckingham, Campbell, Caroline, Charles City, Chesterfield, Clarke, Craig, Culpeper, Cumberland, Dinwiddie, Essex, Fairfax, Fauquier, Fluvanna, Frederick, Giles, Gloucester, Goochland, Greene, Hanover, Henrico, Highland, Isle Of Wight, James City, King And Queen, King George, King William, Lancaster, Loudoun, Louisa, Madison, Mathews, Middlesex, Montgomery, Nelson, New Kent, Northampton, Northumberland, Nottoway, Orange, Page, Powhatan, Prince Edward, Prince George, Prince William, Rappahannock, Richmond, Roanoke, Rockbridge, Rockingham, Shenandoah, Spotsylvania, Stafford, Surry, Warren, Westmoreland, York, Alexandria, Buena Vista, Charlottesville City, Chesapeake City, Colonial Heights City, Covington City, Fairfax City, Falls Church City, Fredericksburg City, Hampton City, Harrisonburg City, Hopewell City, Lexington City, Lynchburg City, Manassas City, Manassas Park City, Newport News City, Norfolk City, Petersburg City, Poquoson City, Portsmouth City, Richmond City, Staunton City, Suffolk City, Virginia Beach City, Waynesboro City, Williamsburg City, Winchester City |
| West Virginia | WV | 11 | Berkeley, Grant, Hampshire, Hardy, Jefferson, Mineral, Monroe, Morgan, Pendleton, Preston, Tucker |

Table S2. Load sources, crop types, and groupings used in this study.

| Sector (Number of Land Sources) | Major Load Source (Number of Land Sources) | Minor Load Source (Number of Land Sources) | Load Source (Number of Related Crops) | Related crops |
| --- | --- | --- | --- | --- |
| Agriculture | Regulated Agriculture | Feeding Space | Permitted Feeding Space |  |
|  | Non-Regulated Agriculture | Row Crops | Double Cropped Land | alfalfa hay, barley for grain, corn for silage or greenchop, other haylage; grass silage and greenchop, rye for grain, small grain hay, sorghum for grain, soybeans for beans, triticale, wheat for grain |
|  |  |  | Full Season Soybeans | soybeans for beans |
|  |  |  | Grain with Manure | corn for grain, sorghum for grain |
|  |  |  | Grain Without Manure | corn for grain, sorghum for grain |
|  |  |  | Other Agronomic Crops | cotton, cropland idle or used for cover crops or soil improvement but not harvested and not pastured or grazed, cropland in cultivated summer fallow, dry edible beans excluding limas, peanuts for nuts, sod, sweet corn, tobacco |
|  |  |  | Silage with Manure | corn for silage or greenchop, sorghum for silage or greenchop |
|  |  |  | Silage Without Manure | corn for silage or greenchop, sorghum for silage or greenchop |
|  |  |  | Small Grains and Grains | barley for grain, buckwheat, canola, emmer and spelt, oats for grain, rye for grain, triticale, wheat for grain |
|  |  |  | Specialty Crop High | bedding/garden plants, beets, broccoli, brussels sprouts, bulbs; corms; rhizomes; and tubers dry, cantaloupe, carrots, cauliflower, celery, chinese cabbage, collards, cucumbers and pickles, cut flowers and cut florist greens, dry onions, eggplant, escarole and endive, foliage plants, garlic, green onions, greenhouse vegetables, head cabbage, herbs - fresh cut, honeydew melons, kale, lettuce, mushrooms, mustard greens, okra, other nursery and greenhouse crops, parsley, peppers - bell, peppers - chile (all peppers excluding bell), popcorn, potatoes, potted flowering plants, pumpkins, radishes, rhubarb, spinach, squash, sweet potatoes, tomatoes, turnip greens, turnips, vegetable & flower seeds, vegetables - mixed, watermelons |
|  |  |  | Specialty Crop Low | aquatic plants, asparagus, berries - all, cut christmas trees production, green lima beans, land in orchards, nursery stock, peas - chinese (sugar and snow), peas - green (excluding southern), peas - green southern (cowpeas), short-rotation woody crops, snap beans, sunflower seed - non-oil varieties, sunflower seed - oil varieties |
|  |  | Riparian Pasture | Riparian Pasture |  |
|  |  | Pasture | Pasture | cropland used only for pasture or grazing, pastureland and rangeland other than cropland and woodland pastured |
|  |  | Feeding Space | Non-Permitted Feeding Space |  |
|  |  | Hay | Leguminous Hay | alfalfa hay, alfalfa seed, birdsfoot trefoil seed, haylage or greenchop from alfalfa or alfalfa mixtures, red clover seed, vetch seed |
|  |  |  | Other Hay | bromegrass seed, cropland on which all crops failed or were abandoned, fescue seed, orchardgrass seed, other field and grass seed crops, other haylage; grass silage and greenchop, other managed hay, ryegrass seed, small grain hay, timothy seed |
|  |  | Other Ag | Ag Open Space | wild hay |
| Developed | Regulated Developed | Construction | CSS Construction |  |
|  |  |  | Regulated Construction |  |
|  |  | Impervious Developed | CSS Buildings and Other |  |
|  |  |  | CSS Roads |  |
|  |  |  | CSS Tree Canopy Over Impervious |  |
|  |  |  | MS4 Buildings and Other |  |
|  |  |  | MS4 Roads |  |
|  |  |  | MS4 Tree Canopy Over Impervious |  |
|  |  | Pervious Developed | CSS Tree Canopy Over Turf Grass | turfgrass |
|  |  |  | CSS Turf Grass | turfgrass |
|  |  |  | MS4 Tree Canopy Over Turf Grass | turfgrass |
|  |  |  | MS4 Turf Grass | turfgrass |
|  | Non-Regulated Developed | Impervious Developed | Non-Regulated Buildings and Other |  |
|  |  |  | Non-Regulated Roads |  |
|  |  |  | Non-Regulated Tree Canopy Over Impervious |  |
|  |  | Pervious Developed | Non-Regulated Tree Canopy Over Turf Grass | turfgrass |
|  |  |  | Non-Regulated Turf Grass | turfgrass |
| Natural | Natural | Forest | CSS Forest |  |
|  |  |  | Harvested Forest |  |
|  |  |  | True Forest |  |
|  |  | Open Space | CSS Mixed Open |  |
|  |  |  | Mixed Open |  |
|  |  | Shoreline | Shoreline |  |
|  |  | Stream | Stream Bed and Bank |  |
|  |  | Wetland | Headwater or Isolated Wetland |  |
|  |  |  | Non-Tidal Floodplain Wetland |  |
|  | Non-Tidal Water Deposition | Non-Tidal Water Deposition | Water |  |
| Septic | Septic and RIB | Septic and RIB | Rapid Infiltration Basin |  |
|  |  |  | Septic |  |
| Wastewater | Combined Sewer Overflow | Combined Sewer Overflow | Combined Sewer Overflow |  |
|  | Wastewater | Wastewater | Industrial Wastewater Treatment Plant |  |

Table S3. Major nitrogen flows included in the CAFE framework by system and their data sources used in this study.

| System | Major nitrogen flows | Definition | Data sources and references |
| --- | --- | --- | --- |
| Cropping system | Fertilizer to row crops (1a-R) | N in fertilizer to row crops | CAST-2019 ^3^ |
|  | N deposition to row crops (1b-R) | N deposition to row crops | CAST-2019 ^3^ |
|  | N fixation by row crops (1c-R) | N fixation by row crops | CAST-2019 ^3^ |
|  | Manure to row crops (2-R) | N in manure to row crops | CAST-2019 ^3^ |
|  | N in non-feed row crops (3-R) | N in non-feed row crops | CAST-2019 ^3^, NANI Accounting Tool V3.1 ^10,11^ |
|  | N in feed row crops to livestock (4-R) | Removal of N in feed row crops to livestock | CAST-2019 ^3^, NANI Accounting Tool V3.1 ^10,11^ |
|  | N loss from domestic consumption recycled to row crops (18a-R) | N loss from domestic consumption recycled to row crops | CAST-2019 ^3^ |
|  | N loss from processing and retail recycled to row crops (19-R) | N loss from processing and retail recycled to row crops | Assumptions, currently assuming 0 |
|  | N loss from Cropping system (14-R) | N loss from Cropping system | Not quantified in this study |
|  | N surplus in Cropping system | Fertilizer to row crops (1a-R) + N deposition to row crops (1b-R) + N fixation by row crops (1c-R) + Manure to row crops (2-R) + N loss from domestic consumption recycled to row crops (18a-R) + N loss from processing and retail recycled to row crops (19-R) - N in non-feed row crops (3-R) - N in feed row crops to livestock (4-R) | Balance |
| Animal-crop system | Fertilizer to other plants (1a-O) | N in fertilizer to other plants (hay + open space) | CAST-2019 ^3^ |
|  | N deposition to other plants (1b-O) | N deposition to other plants | CAST-2019 ^3^ |
|  | N fixation by other plants (1c-O) | N fixation by other plants | CAST-2019 ^3^ |
|  | N in non-feed other plants (3-O) | N in non-feed other plants | CAST-2019 ^3^, NANI Accounting Tool V3.1 ^10,11^ |
|  | N fertilizer to pasture (5a) | N fertilizer to pasture | CAST-2019 ^3^ |
|  | N deposition to pasture (5b) | N deposition to pasture | CAST-2019 ^3^ |
|  | N fixation by grass (5c) | N fixation by grass | CAST-2019 ^3^ |
|  | N manure from animal to pasture (6) | N manure from animal to pasture | CAST-2019 ^3^ |
|  | Net imported feed to animal (8) | Net import (excluding import loss) = N consumed by animal - (1-10%) * (N in feed row crops to livestock (4-R) + N in feed other plants to livestock (4-O)) – N from pasture (7) use 20% loss for hay and silage.  If net import is positive, it is treated as import (8a) and I assume export is 0 (8b); if net import is negative, it is treated as export (8b) and I assume import is 0 (8a). if net import is zero, I assume both import and export are 0. | CAST-2019 ^3^, NANI Accounting Tool V3.1 ^10,11^, Russell, et al. ^16^ |
|  | N in animal production (9) | =N stored in animal = N consumed by animal - N excretion per animal = N in animal products + N in inedible part  N in animal products = N stored in animal * edible portion | See Table S6 |
|  | N loss from domestic consumption recycled to other plants (18a-O) | N loss from domestic consumption recycled to other plants | CAST-2019 ^3^ |
|  | N loss from processing and retail recycled to other plants (19-O) | N loss from processing and retail recycled to other plants | Assumptions, currently assuming 0 |
|  | N loss from domestic consumption recycled to pasture (18b) | N loss from domestic consumption recycled to pasture | CAST-2019 ^3^ |
|  | N loss from Animal-crop system (15) | N loss from Animal-crop system = N loss from pasture (15a) + N loss from animal system (15b) + N loss from other plants (14-O) | Not quantified in this study |
|  | N surplus in Animal-crop system | Fertilizer to row crops (1a-R) + N deposition to row crops (1b-R) + N fixation by row crops (1c-R) + Fertilizer to other plants (1a-O) + N deposition to other plants (1b-O) + N fixation by other plants (1c-O) + Fertilizer to pasture (5a) + N deposition to pasture (5b) + N fixation by grass (5c) + Imported feed (8a) + N loss from domestic consumption recycled to row crops (18a-R) + N loss from domestic consumption recycled to other plants (18a-O) + N loss from domestic consumption recycled to pasture (18b) + N loss from processing and retail recycled to row crops (19-R) + N loss from processing and retail recycled to other crops (19-O) - Exported feed (8b) - N in animal production (9) - N in non-feed row crops (3-R) - N in non-feed other plants (3-O) | Balance |
|  | Surplus increase from Cropping system to Animal-crop system | Fertilizer to other plants (1a-O) + N deposition to other plants (1b-O) + N fixation by other plants (1c-O) + Fertilizer to pasture (5a) + N deposition to pasture (5b) + N fixation by grass (5c) + Imported feed (8a) + N loss from domestic consumption recycled to other plants (18a-O) + N loss from domestic consumption recycled to pasture (18b) + N loss from processing and retail recycled to other crops (19-O) - Exported feed (8b) - N in animal production (9) - N in non-feed other plants (3-O) - Manure to row crops (2-R) + N in feed row crops to livestock (4-R) | Animal-crop system nutrient surplus – Cropping system nutrient surplus |
| Food system | Net imported food, fiber and biofuels = imported food, fiber and biofuels (10) - exported agricultural products (food, fiber, and biofuel) (12) | local production = (1-10%) * N in animal products + (1-10%) * (non-feed row crop production (3-R) + non-feed other plant production (3-O)).  Net import = Agricultural products (food, fiber, and biofuel) for domestic use (11) - local production.  If net import is negative, it is treated as export and import is 0; otherwise, it is treated as import and export is 0. | Russell, et al. ^16^ |
|  | Agricultural products (food, fiber, and biofuel) for domestic use (11) | = estimated consumption rate * population | Sabo, et al. ^17^, NANI Accounting Tool V3.1 ^10,11^, NIH ^19^ |
|  | N loss from processing and retail (16) | N loss from processing and retail | Russell, et al. ^16^ |
|  | N loss from processing and retail recycled to cropland (19) | N loss from processing and retail recycled to cropland | Assumptions, currently assuming 0 |
|  | N surplus in Food system | Fertilizer to row crops (1a-R) + N deposition to row crops (1b-R) + N fixation by row crops (1c-R) + Fertilizer to other plants (1a-O) + N deposition to other plants (1b-O) + N fixation by other plants (1c-O) + Fertilizer to pasture (5a) + N deposition to pasture (5b) + N fixation by grass (5c) + Imported feed (8a) + imported food, fiber and biofuels (10) + N loss from domestic consumption recycled to row crops (18a-R) + N loss from domestic consumption recycled to other plants (18a-O) + N loss from domestic consumption recycled to pasture (18b) - exported agricultural products (food, fiber, and biofuel) (12) - Exported feed (8b) - Agricultural products (food, fiber, and biofuel) for domestic use (11) | Balance |
|  | Surplus increase from Animal-crop system to Food system | imported food, fiber and biofuels (10) - exported agricultural products (food, fiber, and biofuel) (12) - Agricultural products (food, fiber, and biofuel) for domestic use (11) - N loss from processing and retail recycled to row crops (19-R) - N loss from processing and retail recycled to other crops (19-O) + N in animal production (9) + N in non-feed row crops (3-R) + N in non-feed other plants (3-O) | Food system nutrient surplus - Animal-crop system nutrient surplus |
| Ecosystem | N deposition to urban area (13a) | N deposition to urban area | CAST-2019 ^3^ |
|  | N fixation in urban area (13b) | N fixation in urban area | CAST-2019 ^3^ |
|  | N fertilizer in urban area (13c) | N fertilizer in urban area | CAST-2019 ^3^ |
|  | N loss from domestic consumption (17) | N loss from domestic consumption | Not quantified in this study |
|  | N loads from point sources | municipal and industrial wastewater treatment loads, combined sewage overflows, and septic. | CAST-2019 ^3^ |
|  | N surplus in Ecosystem | Fertilizer to row crops (1a-R) + N deposition to row crops (1b-R) + N fixation by row crops (1c-R) + Fertilizer to other plants (1a-O) + N deposition to other plants (1b-O) + N fixation by other plants (1c-O) + Fertilizer to pasture (5a) + N deposition to pasture (5b) + N fixation by grass (5c) + Imported feed (8a) + imported food, fiber and biofuels (10) + N deposition to urban area (13a) + N fixation in urban area (13b) + N fertilizer in urban area (13c) - exported agricultural products (food, fiber, and biofuel) (12) - Exported feed (8b) | Balance |
|  | Surplus increase from Food system to Ecosystem | N deposition to urban area (13a) + N fixation in urban area (13b) + N fertilizer in urban area (13c) - N loss from domestic consumption recycled to row crops (18a-R) - N loss from domestic consumption recycled to other plants (18a-O) - N loss from domestic consumption recycled to pasture (18b) + Agricultural products (food, fiber, and biofuel) for domestic use (11) | Ecosystem nutrient surplus - Food system nutrient surplus |

Table S4. Major phosphorus flows in the CAFE framework by system and their data sources used in this study.

| System | Major phosphorus flows | Definition | Data sources and references |
| --- | --- | --- | --- |
| Cropping system | Fertilizer to row crops (1a-R) | P in fertilizer to row crops | CAST-2019 ^3^ |
|  | P deposition to row crops (1b-R) | P deposition to row crops | CAST-2019 ^3^ |
|  | Manure to row crops (2-R) | P in manure to row crops | CAST-2019 ^3^ |
|  | P in non-feed row crops (3-R) | P in non-feed row crops | CAST-2019 ^3^, NAPI Accounting Tool V3.1 ^10,11^ |
|  | P in feed row crops to livestock (4-R) | P in feed row crops to livestock | CAST-2019 ^3^, NAPI Accounting Tool V3.1 ^10,11^ |
|  | P loss from domestic consumption recycled to row crops (18a-R) | P loss from domestic consumption recycled to row crops | CAST-2019 ^3^ |
|  | P loss from processing and retail recycled to row crops (19-R) | P loss from processing and retail recycled to row crops | Assumptions, currently assuming 0 |
|  | P loss from Cropping system (14-R) | P loss from Cropping system | Not quantified in this study |
|  | P surplus in Cropping system | Fertilizer to row crops (1a-R) + P deposition to row crops (1b-R) + Manure to row crops (2-R) + P loss from domestic consumption recycled to row crops (18a-R) + P loss from processing and retail recycled to row crops (19-R) - P in non-feed row crops (3-R) - P in feed row crops to livestock (4-R) | Balance |
| Animal-crop system | Fertilizer to other plants (1a-O) | P in fertilizer to other plants | CAST-2019 ^3^ |
|  | P deposition to other plants (1b-O) | P deposition to other plants | CAST-2019 ^3^ |
|  | Manure to other plants (2-O) | P in manure to other plants | CAST-2019 ^3^ |
|  | P in feed other plants to livestock (4-O) | P in feed other plants to livestock | CAST-2019 ^3^, NAPI Accounting Tool V3.1 ^10,11^ |
|  | Fertilizer to pasture (5a) | Fertilizer applied to pasture | CAST-2019 ^3^ |
|  | P deposition to pasture (5b) | P deposition to pasture | CAST-2019 ^3^ |
|  | P manure from animal to pasture (6) | P manure from animal to pasture | CAST-2019 ^3^ |
|  | Net imported feed to animal (8) | Net import (excluding import loss) = P consumed by animal - (1-10%) * (P in feed row crops to livestock (4-R) + P in feed other plants to livestock (4-O)) – P from pasture (7).  use 20% loss for hay and silage.  If positive, treat it as import (8a) and assume export is 0 (8b); if negative, treat it as export (8b) and assume import is 0 (8a) | CAST-2019 ^3^, NAPI Accounting Tool V3.1 ^10,11^, Russell, et al. ^16^ |
|  | P in animal production (9) | = P stored in animal = P consumed by animal - P excretion per animal = P in animal products + P in inedible part  P in animal products = P stored in animal * edible portion | See Table S6 |
|  | P loss from domestic consumption recycled to other crops (18a-O) | P loss from domestic consumption recycled to other crops | CAST-2019 ^3^ |
|  | P loss from domestic consumption recycled to pasture (18b) | P loss from domestic consumption recycled to pasture | CAST-2019 ^3^ |
|  | P loss from processing and retail recycled to other crops (19-O) | P loss from processing and retail recycled to other crops | Assumptions, currently assuming 0 |
|  | P loss from other plants (14-O) | P loss from other plants | Not quantified in this study |
|  | P loss from Animal-crop system (15) | P loss from Animal-crop system = P loss from pasture (15a) + P loss from animal system (15b) | Not quantified in this study |
|  | P surplus in Animal-crop system | Fertilizer to row crops (1a-R) + P deposition to row crops (1b-R) + Fertilizer to other plants (1a-O) + P deposition to other plants (1b-O) + Fertilizer to pasture (5a) + P deposition to pasture (5b) + Imported feed (8a) + P loss from domestic consumption recycled to row crops (18a-R) + P loss from domestic consumption recycled to other plants (18a-O) + P loss from domestic consumption recycled to pasture (18b) + P loss from processing and retail recycled to row crops (19-R) + P loss from processing and retail recycled to other plants (19-O) - P in animal production (9) - P in non-feed row crops (3-R) - P in non-feed other plants (3-O)- Exported feed (8b) | Balance |
|  | Surplus increase from Cropping system to Animal-crop system | Fertilizer to other plants (1a-O) + P deposition to other plants (1b-O) + Fertilizer to pasture (5a) + P deposition to pasture (5b) + Imported feed (8a) + P loss from domestic consumption recycled to other plants (18a-O) + P loss from domestic consumption recycled to pasture (18b) + P loss from processing and retail recycled to other plants (19-O) - P in animal production (9) - P in non-feed other plants (3-O)- Exported feed (8b) - Manure to row crops (2-R) + P in feed row crops to livestock (4-R) | Animal-crop system nutrient surplus – Cropping system nutrient surplus |
| Food system | Net imported food, fiber and biofuels = imported food, fiber and biofuels (10) - exported agricultural products (food, fiber, and biofuel) (12) | Net import = Agricultural products (food, fiber, and biofuel) for domestic use (11) - local production.  local production = (1-10%) * P in animal products + (1-10%) * (non-feed row crops production (3-R) + non-feed other plants production (3-O)).  If net import is negative, it is treated as export, and import is 0; otherwise, it is treated as import, and export is 0. | Russell, et al. ^16^ |
|  | Agricultural products (food, fiber, and biofuel) for domestic use (11) | = estimated consumption rate * population | NAPI Accounting Tool V3.1 ^10,11^, Sabo, et al. ^4^, Falcone ^18^, NIH ^19^ |
|  | P loss from processing and retail (16) | P loss in local production and imported products from food processing and retail | Not quantified in this study |
|  | P loss from processing and retail recycled to cropland (19) | P loss from processing and retail recycled to cropland | Assumptions, currently assuming 0 |
|  | P surplus in Food system | Fertilizer to row crops (1a-R) + P deposition to row crops (1b-R) + Fertilizer to other plants (1a-O) + P deposition to other plants (1b-O) + Fertilizer to pasture (5a) + P deposition to pasture (5b) + Imported feed (8a) + imported food, fiber and biofuels (10) + P loss from domestic consumption recycled to row crops (18a-R) + P loss from domestic consumption recycled to other plants (18a-O) + P loss from domestic consumption recycled to pasture (18b) - Exported feed (8b) - Agricultural products (food, fiber, and biofuel) for domestic use (11) - exported agricultural products (food, fiber, and biofuel) (12) | Balance |
|  | Surplus increase from Animal-crop system to Food system | imported food, fiber and biofuels (10) - Agricultural products (food, fiber, and biofuel) for domestic use (11) - exported agricultural products (food, fiber, and biofuel) (12) - P loss from processing and retail recycled to row crops (19-R) - P loss from processing and retail recycled to other plants (19-O) + P in animal production (9) + P in non-feed row crops (3-R) + P in non-feed other plants (3-O) | Food system nutrient surplus - Animal-crop system nutrient surplus |
| Ecosystem | P from rock for non-farm use (13a) | P from rock for non-farm use | CAST-2019 ^3^ |
|  | P deposition to urban area (13b) | P deposition to urban area | CAST-2019 ^3^ |
|  | P loss from domestic consumption (17) | 100% * total P for domestic consumption (11) - P loss from domestic consumption recycled to row crops (18a-R) - P loss from domestic consumption recycled to other plants (18a-O) - P loss from domestic consumption recycled to pasture (18b) (this equation ensures loss >=0) | Not quantified in this study |
|  | P loads from point sources | municipal and industrial wastewater treatment loads, combined sewage overflows, and septic. | CAST-2019 ^3^ |
|  | P surplus in Ecosystem | Fertilizer to row crops (1a-R) + P deposition to row crops (1b-R) + Fertilizer to other plants (1a-O) + P deposition to other plants (1b-O) + Fertilizer to pasture (5a) + P deposition to pasture (5b) + Imported feed (8a) + imported food, fiber and biofuels (10) + P from rock for non-farm use (13a) + P deposition to urban area (13b) - Exported feed (8b) - exported agricultural products (food, fiber, and biofuel) (12) | Balance |
|  | Surplus increase from Food system to Ecosystem | P from rock for non-farm use (13a) + P deposition to urban area (13b) - P loss from domestic consumption recycled to row crops (18a-R) - P loss from domestic consumption recycled to other plants (18a-O) - P loss from domestic consumption recycled to pasture (18b) + Agricultural products (food, fiber, and biofuel) for domestic use (11) | Ecosystem nutrient surplus - Food system nutrient surplus |

Table S5. Crop use parameters to estimate the amount of nutrient used for different purposes. Unit: %. PercToHumanAsFood: ratio of nutrient used as human food. PercToHumanAsNonFood: ratio of nutrient used as human non-food. PercToHumanAsExport: ratio of nutrient exported. PercAsFeed: ratio of nutrient used as feed. PercAsNotUsed: ratio of nutrient used as others.

| LoadSource | CropName | PercToHumanAsFood% | PercToHumanAsNonFood% | PercToHumanAsExport% | PercAsFeed% | PercAsNotUsed% |
| --- | --- | --- | --- | --- | --- | --- |
| Ag_Open_Space | wild_hay | 0 | 0 | 0 | 100 | 0 |
| CSS_Tree_Canopy_over_Turf_Grass | turfgrass | 0 | 0 | 0 | 0 | 100 |
| CSS_Turf_Grass | turfgrass | 0 | 0 | 0 | 0 | 100 |
| Double_Cropped_Land | sorghum_for_grain | 0 | 0 | 0 | 100 | 0 |
| Double_Cropped_Land | alfalfa_hay | 0 | 0 | 0 | 100 | 0 |
| Double_Cropped_Land | barley_for_grain | 3 | 0 | 0 | 97 | 0 |
| Double_Cropped_Land | corn_for_silage_or_greenchop | 0 | 0 | 0 | 100 | 0 |
| Double_Cropped_Land | wheat_for_grain | 61 | 0 | 0 | 39 | 0 |
| Double_Cropped_Land | soybeans_for_beans | 2 | 0 | 0 | 98 | 0 |
| Double_Cropped_Land | other_haylage;_grass_silage_and_greenchop | 0 | 0 | 0 | 100 | 0 |
| Double_Cropped_Land | small_grain_hay | 0 | 0 | 0 | 100 | 0 |
| Double_Cropped_Land | rye_for_grain | 17 | 0 | 0 | 83 | 0 |
| Double_Cropped_Land | triticale | 39 | 0 | 0 | 61 | 0 |
| Full_Season_Soybeans | soybeans_for_beans | 2 | 0 | 0 | 98 | 0 |
| Grain_with_Manure | sorghum_for_grain | 0 | 0 | 0 | 100 | 0 |
| Grain_with_Manure | corn_for_grain | 4 | 0 | 0 | 96 | 0 |
| Grain_without_Manure | sorghum_for_grain | 0 | 0 | 0 | 100 | 0 |
| Grain_without_Manure | corn_for_grain | 4 | 0 | 0 | 96 | 0 |
| Leguminous_Hay | alfalfa_hay | 0 | 0 | 0 | 100 | 0 |
| Leguminous_Hay | red_clover_seed | 0 | 0 | 0 | 100 | 0 |
| Leguminous_Hay | haylage_or_greenchop_from_alfalfa_or_alfalfa_mixtures | 0 | 0 | 0 | 100 | 0 |
| Leguminous_Hay | vetch_seed | 0 | 0 | 0 | 100 | 0 |
| Leguminous_Hay | alfalfa_seed | 0 | 0 | 0 | 100 | 0 |
| Leguminous_Hay | birdsfoot_trefoil_seed | 0 | 0 | 0 | 100 | 0 |
| MS4_Tree_Canopy_over_Turf_Grass | turfgrass | 0 | 0 | 0 | 0 | 100 |
| MS4_Turf_Grass | turfgrass | 0 | 0 | 0 | 0 | 100 |
| non_Regulated_Tree_Canopy_over_Turf_Grass | turfgrass | 0 | 0 | 0 | 0 | 100 |
| non_Regulated_Turf_Grass | turfgrass | 0 | 0 | 0 | 0 | 100 |
| Other_Agronomic_Crops | tobacco | 0 | 0 | 100 | 0 | 0 |
| Other_Agronomic_Crops | cropland_idle_or_used_for_cover_crops_or_soil_improvement_but_not_harvested_and_not_pastured_or_grazed | 100 | 0 | 0 | 0 | 0 |
| Other_Agronomic_Crops | sweet_corn | 4 | 0 | 0 | 96 | 0 |
| Other_Agronomic_Crops | sod | 100 | 0 | 0 | 0 | 0 |
| Other_Agronomic_Crops | cropland_in_cultivated_summer_fallow | 100 | 0 | 0 | 0 | 0 |
| Other_Agronomic_Crops | dry_edible_beans_excluding_limas | 100 | 0 | 0 | 0 | 0 |
| Other_Agronomic_Crops | cotton | 0 | 0 | 100 | 0 | 0 |
| Other_Agronomic_Crops | peanuts_for_nuts | 100 | 0 | 0 | 0 | 0 |
| Other_Hay | timothy_seed | 0 | 0 | 0 | 100 | 0 |
| Other_Hay | other_managed_hay | 0 | 0 | 0 | 100 | 0 |
| Other_Hay | cropland_on_which_all_crops_failed_or_were_abandoned | 0 | 0 | 0 | 100 | 0 |
| Other_Hay | other_field_and_grass_seed_crops | 0 | 0 | 0 | 100 | 0 |
| Other_Hay | other_haylage;_grass_silage_and_greenchop | 0 | 0 | 0 | 100 | 0 |
| Other_Hay | small_grain_hay | 0 | 0 | 0 | 100 | 0 |
| Other_Hay | orchardgrass_seed | 0 | 0 | 0 | 100 | 0 |
| Other_Hay | ryegrass_seed | 0 | 0 | 0 | 100 | 0 |
| Other_Hay | fescue_seed | 0 | 0 | 0 | 100 | 0 |
| Pasture | cropland_used_only_for_pasture_or_grazing | 0 | 0 | 0 | 100 | 0 |
| Pasture | pastureland_and_rangeland_other_than_cropland_and_woodland_pastured | 0 | 0 | 0 | 100 | 0 |
| Silage_with_Manure | corn_for_silage_or_greenchop | 0 | 0 | 0 | 100 | 0 |
| Silage_with_Manure | sorghum_for_silage_or_greenchop | 0 | 0 | 0 | 100 | 0 |
| Silage_without_Manure | corn_for_silage_or_greenchop | 0 | 0 | 0 | 100 | 0 |
| Silage_without_Manure | sorghum_for_silage_or_greenchop | 0 | 0 | 0 | 100 | 0 |
| Small_Grains_and_Grains | oats_for_grain | 6 | 0 | 0 | 94 | 0 |
| Small_Grains_and_Grains | buckwheat | 61 | 0 | 0 | 39 | 0 |
| Small_Grains_and_Grains | rye_for_grain | 17 | 0 | 0 | 83 | 0 |
| Small_Grains_and_Grains | barley_for_grain | 3 | 0 | 0 | 97 | 0 |
| Small_Grains_and_Grains | wheat_for_grain | 61 | 0 | 0 | 39 | 0 |
| Small_Grains_and_Grains | triticale | 39 | 0 | 0 | 61 | 0 |
| Small_Grains_and_Grains | canola | 100 | 0 | 0 | 0 | 0 |
| Small_Grains_and_Grains | emmer_and_spelt | 61 | 0 | 0 | 39 | 0 |
| Specialty_Crop_High | eggplant | 100 | 0 | 0 | 0 | 0 |
| Specialty_Crop_High | mushrooms | 100 | 0 | 0 | 0 | 0 |
| Specialty_Crop_High | okra | 100 | 0 | 0 | 0 | 0 |
| Specialty_Crop_High | peppers_-_bell | 100 | 0 | 0 | 0 | 0 |
| Specialty_Crop_High | potatoes | 100 | 0 | 0 | 0 | 0 |
| Specialty_Crop_High | radishes | 100 | 0 | 0 | 0 | 0 |
| Specialty_Crop_High | broccoli | 100 | 0 | 0 | 0 | 0 |
| Specialty_Crop_High | escarole_and_endive | 100 | 0 | 0 | 0 | 0 |
| Specialty_Crop_High | potted_flowering_plants | 0 | 100 | 0 | 0 | 0 |
| Specialty_Crop_High | vegetables_-_mixed | 100 | 0 | 0 | 0 | 0 |
| Specialty_Crop_High | foliage_plants | 0 | 100 | 0 | 0 | 0 |
| Specialty_Crop_High | kale | 100 | 0 | 0 | 0 | 0 |
| Specialty_Crop_High | spinach | 100 | 0 | 0 | 0 | 0 |
| Specialty_Crop_High | tomatoes | 100 | 0 | 0 | 0 | 0 |
| Specialty_Crop_High | carrots | 100 | 0 | 0 | 0 | 0 |
| Specialty_Crop_High | celery | 100 | 0 | 0 | 0 | 0 |
| Specialty_Crop_High | mustard_greens | 100 | 0 | 0 | 0 | 0 |
| Specialty_Crop_High | other_nursery_and_greenhouse_crops | 100 | 0 | 0 | 0 | 0 |
| Specialty_Crop_High | pumpkins | 100 | 0 | 0 | 0 | 0 |
| Specialty_Crop_High | sweet_potatoes | 100 | 0 | 0 | 0 | 0 |
| Specialty_Crop_High | brussels_sprouts | 100 | 0 | 0 | 0 | 0 |
| Specialty_Crop_High | bulbs; corms;_rhizomes;_and_tubers_dry | 100 | 0 | 0 | 0 | 0 |
| Specialty_Crop_High | bedding/garden_plants | 100 | 0 | 0 | 0 | 0 |
| Specialty_Crop_High | greenhouse_vegetables | 100 | 0 | 0 | 0 | 0 |
| Specialty_Crop_High | head_cabbage | 100 | 0 | 0 | 0 | 0 |
| Specialty_Crop_High | turnips | 100 | 0 | 0 | 0 | 0 |
| Specialty_Crop_High | beets | 100 | 0 | 0 | 0 | 0 |
| Specialty_Crop_High | chinese_cabbage | 100 | 0 | 0 | 0 | 0 |
| Specialty_Crop_High | cucumbers_and_pickles | 100 | 0 | 0 | 0 | 0 |
| Specialty_Crop_High | cut_flowers_and_cut_florist_greens | 0 | 100 | 0 | 0 | 0 |
| Specialty_Crop_High | squash | 100 | 0 | 0 | 0 | 0 |
| Specialty_Crop_High | cauliflower | 100 | 0 | 0 | 0 | 0 |
| Specialty_Crop_High | honeydew_melons | 100 | 0 | 0 | 0 | 0 |
| Specialty_Crop_High | collards | 100 | 0 | 0 | 0 | 0 |
| Specialty_Crop_High | dry_onions | 100 | 0 | 0 | 0 | 0 |
| Specialty_Crop_High | green_onions | 100 | 0 | 0 | 0 | 0 |
| Specialty_Crop_High | parsley | 100 | 0 | 0 | 0 | 0 |
| Specialty_Crop_High | vegetable_&_flower_seeds | 100 | 0 | 0 | 0 | 0 |
| Specialty_Crop_High | watermelons | 100 | 0 | 0 | 0 | 0 |
| Specialty_Crop_High | cantaloupe | 100 | 0 | 0 | 0 | 0 |
| Specialty_Crop_High | herbs_-_fresh_cut | 100 | 0 | 0 | 0 | 0 |
| Specialty_Crop_High | lettuce | 100 | 0 | 0 | 0 | 0 |
| Specialty_Crop_High | peppers_-_chile_(all_peppers_ _excluding_bell) | 100 | 0 | 0 | 0 | 0 |
| Specialty_Crop_High | garlic | 100 | 0 | 0 | 0 | 0 |
| Specialty_Crop_High | rhubarb | 100 | 0 | 0 | 0 | 0 |
| Specialty_Crop_High | popcorn | 100 | 0 | 0 | 0 | 0 |
| Specialty_Crop_High | turnip_greens | 100 | 0 | 0 | 0 | 0 |
| Specialty_Crop_Low | sunflower_seed_-_non-oil_varieties | 100 | 0 | 0 | 0 | 0 |
| Specialty_Crop_Low | snap_beans | 100 | 0 | 0 | 0 | 0 |
| Specialty_Crop_Low | berries_-_all | 100 | 0 | 0 | 0 | 0 |
| Specialty_Crop_Low | cut_christmas_trees_production | 0 | 100 | 0 | 0 | 0 |
| Specialty_Crop_Low | peas_-_green_(excluding_southern) | 100 | 0 | 0 | 0 | 0 |
| Specialty_Crop_Low | aquatic_plants | 100 | 0 | 0 | 0 | 0 |
| Specialty_Crop_Low | nursery_stock | 0 | 100 | 0 | 0 | 0 |
| Specialty_Crop_Low | green_lima_beans | 100 | 0 | 0 | 0 | 0 |
| Specialty_Crop_Low | peas_-_green_southern_(cowpeas) | 100 | 0 | 0 | 0 | 0 |
| Specialty_Crop_Low | peas_-_chinese_(sugar_and_snow) | 100 | 0 | 0 | 0 | 0 |
| Specialty_Crop_Low | asparagus | 100 | 0 | 0 | 0 | 0 |
| Specialty_Crop_Low | land_in_orchards | 100 | 0 | 0 | 0 | 0 |
| Specialty_Crop_Low | sunflower_seed_-_oil_varieties | 100 | 0 | 0 | 0 | 0 |
| Specialty_Crop_Low | short-rotation_woody_crops | 0 | 100 | 0 | 0 | 0 |

Table S6. Animal parameters used in this study. Parameters were calculated based on excretion and production parameters from Le Noë, et al. ^13^, Le Noe ^49^, Sabo, et al. ^17^, and Russell, et al. ^16^.

| Animal name | Nutrient type | Nutrient excretion to nutrient intake ratio (%) | Nutrient edible portion to nutrient production ratio (%) |
| --- | --- | --- | --- |
| beef | Nitrogen | 87.7 | 45.6 |
| beef | Phosphorus | 62.5 | 10.8 |
| broilers | Nitrogen | 53.8 | 42.8 |
| broilers | Phosphorus | 60.6 | 6.8 |
| dairy | Nitrogen | 77.6 | 100.0 |
| dairy | Phosphorus | 69.0 | 69.0 |
| goats | Nitrogen | 83.8 | 45.3 |
| goats | Phosphorus | 65.9 | 13.2 |
| hogs_and_pigs_for_breeding | Nitrogen | 68.6 | 59.2 |
| hogs_and_pigs_for_breeding | Phosphorus | 73.0 | 16.9 |
| hogs_for_slaughter | Nitrogen | 68.6 | 59.2 |
| hogs_for_slaughter | Phosphorus | 73.0 | 16.9 |
| horses | Nitrogen | 89.3 | 0.0 |
| horses | Phosphorus | 60.0 | 0.0 |
| layers | Nitrogen | 65.5 | 50.1 |
| layers | Phosphorus | 90.0 | 74.4 |
| other_cattle | Nitrogen | 87.7 | 45.6 |
| other_cattle | Phosphorus | 62.5 | 10.8 |
| pullets | Nitrogen | 53.8 | 42.8 |
| pullets | Phosphorus | 88.9 | 6.8 |
| sheep_and_lambs | Nitrogen | 83.8 | 45.3 |
| sheep_and_lambs | Phosphorus | 65.9 | 13.2 |
| turkeys | Nitrogen | 62.9 | 42.8 |
| turkeys | Phosphorus | 78.1 | 6.8 |

Table S7. Summary of parameters used to estimate dishwasher detergent consumption in kgP per capita per year. Parameters are picked by state and year based on Han, et al. ^50^ and Sabo, et al. ^4^, and state-level phosphate detergent ban information is from Litke ^51^.

| State/district | 1985-1986 | 1987-1991 | 1992-2010 | 2011-2019 |
| --- | --- | --- | --- | --- |
| Delaware | 0.5 | 0.74 | 0.5 | 0.5 |
| West Virginia | 0.5 | 0.74 | 0.5 | 0.5 |
| DC | 0.5 | 0.74 | 0.5 | 0.1643 |
| Maryland | 0.5 | 0.74 | 0.5 | 0.1643 |
| New York | 0.5 | 0.74 | 0.5 | 0.1643 |
| Pennsylvania | 0.5 | 0.74 | 0.5 | 0.1643 |
| Virginia | 0.5 | 0.74 | 0.5 | 0.1643 |

Table S8. Summary of parameters used to estimate laundry detergent consumption in kgP per capita per year. Parameters are picked by state and year based on van Puijenbroek, et al. ^52^, and state-level phosphate detergent ban information is from Litke ^51^.

| State/district | 1986 | 1986 | 1987 | 1988 | 1989 | 1990 | 1991-2009 | 2010-2019 |
| --- | --- | --- | --- | --- | --- | --- | --- | --- |
| Delaware | 0.24 | 0.24 | 0.24 | 0.24 | 0.24 | 0.24 | 0.24 | 0.1 |
| West Virginia | 0.24 | 0.24 | 0.24 | 0.24 | 0.24 | 0.24 | 0.24 | 0.1 |
| DC | 0.24 | 0.24 | 0 | 0 | 0 | 0 | 0 | 0 |
| Maryland | 0.24 | 0 | 0 | 0 | 0 | 0 | 0 | 0 |
| New York | 0 | 0 | 0 | 0 | 0 | 0 | 0 | 0 |
| Pennsylvania | 0.24 | 0.24 | 0.24 | 0.24 | 0.24 | 0.24 | 0 | 0 |
| Virginia | 0.24 | 0.24 | 0.24 | 0.24 | 0 | 0 | 0 | 0 |

# References

1 Zhang, X. *et al.* Quantifying nutrient budgets for sustainable nutrient management. *Global Biogeochemical Cycles* **34**, doi:10.1029/2018gb006060 (2020).

2 Li, T. *et al.* A hierarchical framework for unpacking the nitrogen challenge. *Earth's Future* **10**, e2022EF002870, doi:10.1029/2022EF002870 (2022).

3 CBP. *Chesapeake assessment and scenario tool (cast) version 2019*, <<https://cast.chesapeakebay.net/about>> (2020), Last accessed 2020.

4 Sabo, R. D. *et al.* Phosphorus inventory for the conterminous united states (2002–2012). *Journal of Geophysical Research: Biogeosciences* **126**, e2020JG005684, doi:10.1029/2020JG005684 (2021).

5 Sabo, R. D. *et al.* Major point and nonpoint sources of nutrient pollution to surface water have declined throughout the chesapeake bay watershed. *Environmental Research Communications* **4**, 045012, doi:10.1088/2515-7620/ac5db6 (2022).

6 Keisman, J. L. D., Devereux, O. H., LaMotte, A. E., Sekellick, A. J. & Blomquist, J. D. Manure and fertilizer inputs to land in the chesapeake bay watershed, 1950–2012. (<<https://doi.org/10.3133/sir20185022>> (2018).

7 Zhang, X. *et al.* Managing nitrogen for sustainable development. *Nature* **528**, 51-59, doi:10.1038/nature15743 (2015).

8 Zou, T., Zhang, X. & Davidson, E. A. Global trends of cropland phosphorus use and sustainability challenges. *Nature* **611**, 81-87, doi:10.1038/s41586-022-05220-z (2022).

9 Zhang, X. *et al.* Quantification of global and national nitrogen budgets for crop production. *Nature Food* **2**, 529-540, doi:10.1038/s43016-021-00318-5 (2021).

10 Hong, B., Swaney, D. P. & Howarth, R. W. A toolbox for calculating net anthropogenic nitrogen inputs (nani). *Environmental Modelling & Software* **26**, 623-633, doi:10.1016/j.envsoft.2010.11.012 (2011).

11 Swaney, D. P., Hong, B. & Howarth, R. W. *Nani/napi calculator toolbox version 3.1 documentation*, <<http://www.eeb.cornell.edu/biogeo/nanc/nani/NANINAPI_Calculator_Toolbox_Version_3.1_Documentation.docx>> (2018), Last accessed 2020.

12 USCensusBureau. *USA counties: 2011*, <<https://www.census.gov/library/publications/2011/compendia/usa-counties-2011.html#LND>> (2011), Last accessed 2021.

13 Le Noë, J., Billen, G. & Garnier, J. How the structure of agro-food systems shapes nitrogen, phosphorus, and carbon fluxes: The generalized representation of agro-food system applied at the regional scale in france. *Science of The Total Environment* **586**, 42-55, doi:10.1016/j.scitotenv.2017.02.040 (2017).

14 Whiting, T. L. The united states' prohibition of horsemeat for human consumption: Is this a good law? *Can Vet J* **48**, 1173-1180 (2007).

15 Wang, C. *et al.* Effects of dietary supplementation of methionine and lysine on milk production and nitrogen utilization in dairy cows1. *Journal of Dairy Science* **93**, 3661-3670, doi:10.3168/jds.2009-2750 (2010).

16 Russell, M. J., Weller, D. E., Jordan, T. E., Sigwart, K. J. & Sullivan, K. J. Net anthropogenic phosphorus inputs: Spatial and temporal variability in the chesapeake bay region. *Biogeochemistry* **88**, 285-304, doi:10.1007/s10533-008-9212-9 (2008).

17 Sabo, R. D. *et al.* Decadal shift in nitrogen inputs and fluxes across the contiguous united states: 2002–2012. *Journal of Geophysical Research: Biogeosciences* **124**, 3104-3124, doi:10.1029/2019jg005110 (2019).

18 Falcone, J. *Us block-level population density rasters for 1990, 2000, and 2010*, <<https://data.usgs.gov/datacatalog/data/USGS:57753ebee4b07dd077c70868>> (2016), Last accessed 2022.

19 NIH. *Download u.S. County population data - 1969-2020*, <<https://seer.cancer.gov/popdata/download.html#19>> (2021), Last accessed 2021.

20 Sabo, R. D., Clark, C. M. & Compton, J. E. Considerations when using nutrient inventories to prioritize water quality improvement efforts across the us. *Environmental Research Communications* **3**, 045005, doi:10.1088/2515-7620/abf296 (2021).

21 CBC. The chesapeake bay and its watershed. (Chesapeake Bay Commission <<https://www.chesbay.us/library/public/documents/Fact-Sheets/Bay-Factoids-FINAL.pdf>> (2020).

22 CBF. *The history of chesapeake bay cleanup efforts - chesapeake bay foundation*, <<https://www.cbf.org/how-we-save-the-bay/chesapeake-clean-water-blueprint/the-history-of-bay-cleanup-efforts.html>> (2023), Last accessed 2023.

23 Krikstan, C. *Chesapeake bay program partnership exceeds 2017 pollution reducing targets for phosphorus, sediment*, <<https://www.chesapeakebay.net/news/blog/partnership-exceeds-2017-pollution-reducing-targets-for-phosphorus-sediment>> (2018), Last accessed 2022.

24 Lingenfelter, D. *Nutrient management legislation in pennsylvania*, <<https://extension.psu.edu/nutrient-management-legislation-in-pennsylvania>> (2014), Last accessed 2023.

25 Sims, J. T., Shober, A. L., Clark, K. L., Leytem, A. B. & Coale, F. J. The delaware phosphorus site index technical guidance manual (University of Delaware Cooperative Extension, <<https://www.udel.edu/content/dam/udelImages/canr/pdfs/extension/factsheets/P-Site-Index-Technical-Guide-Manual.pdf>> (2016).

26 USEPA. *Chesapeake bay tributary strategies*, <<https://www.epa.gov/chesapeake-bay-tmdl/chesapeake-bay-tributary-strategies>> (2021), Last accessed 2023.

27 Kleinman, P. J. A. *et al.* Phosphorus and the chesapeake bay: Lingering issues and emerging concerns for agriculture. *Journal of Environmental Quality* **48**, 1191-1203, doi:10.2134/jeq2019.03.0112 (2019).

28 Chang, S. Y., Zhang, Q., Byrnes, D. K., Basu, N. B. & Van Meter, K. J. Chesapeake legacies: The importance of legacy nitrogen to improving chesapeake bay water quality. *Environmental Research Letters* **16**, 085002, doi:10.1088/1748-9326/ac0d7b (2021).

29 Pomar, C., Andretta, I. & Remus, A. Feeding strategies to reduce nutrient losses and improve the sustainability of growing pigs. *Front Vet Sci* **8**, 742220, doi:10.3389/fvets.2021.742220 (2021).

30 Dora, M. *et al.* Importance of sustainable operations in food loss: Evidence from the belgian food processing industry. *Annals of Operations Research* **290**, 47-72, doi:10.1007/s10479-019-03134-0 (2020).

31 Lipinski, B., Hanson, C., Waite, R., Searchinger, T. & Lomax, J. Reducing food loss and waste. (World Resources Institute, 2013).

32 Cordell, D., Drangert, J.-O. & White, S. The story of phosphorus: Global food security and food for thought. *Global Environmental Change* **19**, 292-305, doi:10.1016/j.gloenvcha.2008.10.009 (2009).

33 Spiker, M. L., Hiza, H. A. B., Siddiqi, S. M. & Neff, R. A. Wasted food, wasted nutrients: Nutrient loss from wasted food in the united states and comparison to gaps in dietary intake. *Journal of the Academy of Nutrition and Dietetics* **117**, 1031-1040.e1022, doi:10.1016/j.jand.2017.03.015 (2017).

34 Pimental, A. *Maryland passes law to reduce pollution from lawn fertilizer*, <<https://www.chesapeakebay.net/news/blog/maryland-passes-law-to-reduce-pollution-from-lawn-fertilizer>> (2011), Last accessed 2022.

35 MacDonald, G. K., Bennett, E. M. & Carpenter, S. R. Embodied phosphorus and the global connections of united states agriculture. *Environmental Research Letters* **7**, 044024, doi:10.1088/1748-9326/7/4/044024 (2012).

36 Kenny, S., Stephenson, J., Stern, A. & Beecher, J. From field to bin: The environmental impacts of u.S. Food waste management pathways (part 2). (U.S. Environmental Protection Agency, 2023).

37 Kanter, D. R. *et al.* Nitrogen pollution policy beyond the farm. *Nature Food* **1**, 27-32, doi:10.1038/s43016-019-0001-5 (2020).

38 Spiegal, S. *et al.* Manuresheds: Advancing nutrient recycling in us agriculture. *Agricultural Systems* **182**, 102813, doi:10.1016/j.agsy.2020.102813 (2020).

39 Powers, S. M. *et al.* Global opportunities to increase agricultural independence through phosphorus recycling. *Earth's Future* **7**, 370-383, doi:10.1029/2018EF001097 (2019).

40 Kleinman, P. J. A. *et al.* in *Animal manure* *Asa special publications* 201-228 (2020).

41 Metson, G. S., MacDonald, G. K., Haberman, D., Nesme, T. & Bennett, E. M. Feeding the corn belt: Opportunities for phosphorus recycling in u.S. Agriculture. *Science of The Total Environment* **542**, 1117-1126, doi:10.1016/j.scitotenv.2015.08.047 (2016).

42 Pozzebon, E. A. & Seifert, L. Emerging environmental health risks associated with the land application of biosolids: A scoping review. *Environmental Health* **22**, 57, doi:10.1186/s12940-023-01008-4 (2023).

43 Leib, E. B. *et al.* Leftovers for livestock: A legal guide for using excess food as animal feed. (The Harvard Food Law and Policy Clinic and the Food Recovery Project at the University of Arkansas School of Law, 2016).

44 Margenot, A. J. *et al.* Toward a regional phosphorus (re)cycle in the us midwest. *Journal of Environmental Quality* **48**, 1397-1413, doi:10.2134/jeq2019.02.0068 (2019).

45 USEPA. *United states 2030 food loss and waste reduction goal*, <<https://www.epa.gov/sustainable-management-food/united-states-2030-food-loss-and-waste-reduction-goal#:~:text=The%202030%20FLW%20reduction%20goal%20aims%20to%20reduce%20food%20waste,to%20109.4%20pounds%20per%20person.&text=In%20the%20baseline%20year%20of,estimated%20value%20of%20%24161.6%20billion>.> (2020), Last accessed 2022.

46 Yadav, S. *et al.* Updated review on emerging technologies for pfas contaminated water treatment. *Chemical Engineering Research and Design* **182**, 667-700, doi:10.1016/j.cherd.2022.04.009 (2022).

47 USEPA. *Key epa actions to address pfas*, <<https://www.epa.gov/pfas/key-epa-actions-address-pfas>> (2024), Last accessed 2024.

48 Kruskal, W. H. & Wallis, W. A. Use of ranks in one-criterion variance analysis. *Journal of the American Statistical Association* **47**, 583-621, doi:10.1080/01621459.1952.10483441 (1952).

49 Le Noe, J. *Biogeochemical functioning and trajectories of french territorial agricultural systems. Carbon, nitrogen and phosphorus fluxes (1852-2014)* PhD thesis, University Pierre and Marie Curie, (2018).

50 Han, H., Bosch, N. & Allan, J. D. Spatial and temporal variation in phosphorus budgets for 24 watersheds in the lake erie and lake michigan basins. *Biogeochemistry* **102**, 45-58, doi:10.1007/s10533-010-9420-y (2011).

51 Litke, D. W. *Review of phosphorus control measures in the united states and their effects on water quality*. Vol. 99 (US Department of the Interior, US Geological Survey, 1999).

52 van Puijenbroek, P. J. T. M., Beusen, A. H. W. & Bouwman, A. F. Datasets of the phosphorus content in laundry and dishwasher detergents. *Data in Brief* **21**, 2284-2289, doi:10.1016/j.dib.2018.11.081 (2018).
